# Force variability is mostly not motor noise: Theoretical implications for motor control

**DOI:** 10.1371/journal.pcbi.1008707

**Published:** 2021-03-08

**Authors:** Akira Nagamori, Christopher M. Laine, Gerald E. Loeb, Francisco J. Valero-Cuevas

**Affiliations:** 1 Division of Biokinesiology and Physical Therapy, University of Southern California, Los Angeles, California, United States of America; 2 Chan Division of Occupational Science and Occupational Therapy, University of Southern California, Los Angeles, California, United States of America; 3 Department of Biomedical Engineering, University of Southern California, Los Angeles, California, United States of America; Imperial College London, UNITED KINGDOM

## Abstract

Variability in muscle force is a hallmark of healthy and pathological human behavior. Predominant theories of sensorimotor control assume ‘motor noise’ leads to force variability and its ‘signal dependence’ (variability in muscle force whose amplitude increases with intensity of neural drive). Here, we demonstrate that the two proposed mechanisms for motor noise (i.e. the stochastic nature of motor unit discharge and unfused tetanic contraction) cannot account for the majority of force variability nor for its signal dependence. We do so by considering three previously underappreciated but physiologically important features of a population of motor units: 1) fusion of motor unit twitches, 2) coupling among motoneuron discharge rate, cross-bridge dynamics, and muscle mechanics, and 3) a series-elastic element to account for the aponeurosis and tendon. These results argue strongly against the idea that force variability and the resulting kinematic variability are generated primarily by ‘motor noise.’ Rather, they underscore the importance of variability arising from properties of control strategies embodied through distributed sensorimotor systems. As such, our study provides a critical path toward developing theories and models of sensorimotor control that provide a physiologically valid and clinically useful understanding of healthy and pathologic force variability.

## Author summary

Variability in our movements is thought to arise predominantly from ‘noise’ in the processes that convert central neural drive into muscle force. Constant variance of such noise has been a theoretical basis from which to explain various aspects of motor behavior. However, the physiological basis for such an assumption has not been tested rigorously. Our new computational model of a population of motor units demonstrates that non-physiological assumptions in previous models have led to erroneous interpretations of the role and significance of motor unit properties in the generation of force variability. Our results provide a clear path forward for future efforts using computational modeling to build theories of how altered neuromuscular systems emerge in aging or neurological disorders.

## Introduction

Variability is a hallmark of healthy and pathological human behavior. As such, the structure of kinematic [1] and kinetic variability [2, 3] is a rich behavioral phenomenon that informs theoretical models about the mechanisms used by the central nervous system to learn and produce motor behaviors [4–14]. These theories are then used to help identify the mechanisms underlying dysfunction and features of various neurological conditions [15, 16]. Therefore, the physiological validity of these theoretical models is critical to their proper and effective clinical translation.

Many theoretical models for human motor behavior (e.g. minimum variance theory and optimal control theory) assume that observed kinematic variability arises predominantly, if not exclusively, from ‘signal-dependent motor noise;’ random variations in muscle force output whose amplitude increases with the input level [5–9, 11–14, 17, 18]. Under this theoretical framework, observed kinematics and its variability reflect the performance limitation imposed by the motor system [6, 18] attempting to minimize the deleterious effects of ‘motor noise’ on behavior [12, 17]. In these models, the specific implementations of motor noise (i.e. amplitude and its relationship with input levels) are convenient free parameters that determine model performance and allow fitting of model output to experimental data. Furthermore, many clinical manifestations of increased force/kinematic variability have been interpreted simply as a consequence of increased motor noise interfering with optimal control strategies implemented by the central nervous system [15, 19, 20].

Despite the success of these theoretical models replicating certain experimental observations, their theoretical framework is incompatible with many other experimental observations. For one, they cannot explain why force/kinematic variability can be modified by various factors such as visual feedback and physical activity [21, 22], how such variability can be tuned as needed to meet task demands and to enhance sensing or exploration [4], or why the amplitude and spectral structure of force variability is so heavily dependent on closed-loop elements of force control, such as segmental and visiomotor feedback loops [21, 23–26].

Secondly, the physiological basis for ‘motor noise’ is very weak. It has been assumed that motor noise arises from the physiological properties of motor units, in particular, stochastic timing of motor unit discharges and unfused tetanic contraction [6, 27–29]. This view is supported by many simulations based upon the seminal computational model of motor unit recruitment and rate coding originally proposed by Fuglevand et al. [30]. Despite many successful applications of the Fuglevand model [9, 28, 31–41], its usage to explain the origin of force variability is, in fact, an extrapolation of its original intent and scope, which is to simulate the relationship between isometric force and electromyographic activity of muscle. It has been extensively re-purposed and modified to fit experimentally observed amplitude of force variability and its signal dependence, despite containing various non-physiological motor unit features/assumptions that may render it inappropriate for this purpose. As a result, some studies may have overstated the significance of motor unit properties in shaping experimentally observed signal-dependent noise during isometric contractions (e.g. Jones et al. [28]). Such overstatement promoted the emergence of a premature consensus in the community that force variability arises mostly or exclusively from motor noise (e.g. Todorov [12]). This interpretation is incompatible with early and recent experimental observations that closed-loop elements of force control (e.g. such as segmental and visuomotor feedback loops) can alter the amplitude and spectral structure of force variability [21, 23–25, 42].

In this study, we systematically examine the physiological validity of the assumptions that underlie various motor unit models and their implications to force variability. To this end, we developed a new model of a population of motor units that now includes three physiologically important features: 1) calcium kinetics and cross-bridge dynamics that drives fusion of motor unit twitches, 2) coupling among motoneuron discharge rate, cross-bridge dynamics, and muscle mechanics, and 3) a series-elastic element to account for the aponeurosis and tendon. By exploring a plausible range of parameters for these known physiological processes, we were able to test the following two hypotheses: A) Those model refinements significantly reduce the amplitude of ‘motor noise’ and its significance in shaping overall force variability, and B) The refined model output directly contradicts the assumption that motor noise should increase continually with force (i.e. is ‘signal dependent’, as typically defined), because the completeness of twitch fusion at higher levels of synaptic input should increase.

Our results support the above two hypotheses, demonstrating that experimentally observed amplitude of force variability and its signal dependence cannot be explained by motor noise alone. Therefore, our results argue strongly against the idea that force variability should be modeled as the consequence of ‘motor noise.’ Rather, our results emphasize the importance of alternative sources of force variability arising from control strategies embodied through distributed sensorimotor systems, which are underestimated or ignored in current models of motor behavior. Therefore, our study informs fruitful directions to better understand and interpret force variability in health and disease [31, 43–45].

## Materials and methods

### Conversion of spike trains into motor unit force using Fuglevand model

The model of rate coding and recruitment of a motor unit pool developed by Fuglevand et al. [30] was replicated and tested against known physiological properties of motor units. Only brief descriptions of this model are provided here, as it is fully described in the original paper [30]. Motor unit force is modeled as the impulse response of a second-order critically damped system as in the following equation [30]:

$$\begin{array}{cc} f_{i}\left(t\right)=g_{i,j}\frac{P_{i}t}{T_{i}}e^{1-(t/T_{i})}, & \end{array}$$ (1)

where *g*, *P*, *T* and *t* are the gain, peak twitch force, contraction time and time, respectively. The subscripts, *i* and *j*, denote the indexes of motor units and of motor unit spike events. The gain, *g*_*i*,*j*_, was originally introduced to replicate the sigmoidal relationship between the discharge rate of a motor unit and its output force as described in Eq. 17 in [30]. The peak twitch force, *P*_*i*_, and contraction time, *T*_*i*_, of each motor unit follow a exponential distribution with a specified range between the smallest (slowest) and largest (fastest) motor units. The original model proposed in [30] uses 100-fold and 3-fold ranges for the peak twitch force and contraction time, respectively, which were used here. Similarly, the recruitment threshold of motor units follows an exponential distribution such that the range of lowest- and highest-threshold units equals to a 68-fold difference in an excitation unit, *E*, in the original model. Combined with the value of *g*_*e*_ = 1, this corresponds to the recruitment of all motor units at 80% of the maximal excitation, which has been reported for the *tibialis anterior* muscle [46, 47].

### New model of a motor unit pool

Despite its original purpose for simulating the relationship between isometric muscle force and electromyogram (EMG) of muscle [30], the Fuglevand model has been repurposed to simulate force variability [9, 28, 31–41, 48]. However, such use of the Fuglevand model has several critical drawbacks that limit its physiological faithfulness to simulate force variability as described in detail below:

1. The peak tetanic force of motor units, and therefore that of muscle, depend on an arbitrary choice of model parameters such as the peak discharge rate of a given unit. We found this non-physiological because the determinants of the maximal force of a given unit are the number of muscle fibers (i.e. innervation ratio), the muscle fibers cross-sectional area, and their specific tension [49].
2. The simulated range of discharge rates for a given motor unit was based on over-simplification and idealization of empirical observations from human motor units, which do not always reflect current theoretical and functional understanding of muscle.
3. The model does not explicitly simulate the fusion of force twitches with increases in discharge rate and the concomitant saturation of calcium binding to troponin. In fact, the model does not always produce the fusion of force twitches.
4. The model lacks a series elastic element (i.e. tendon and aponeurosis). Even during the isometric condition it was originally intended to simulate, muscle length fluctuates due to this in-series compliance. Such muscle length fluctuations inevitably affects the viscoelastic properties of force generation, which can significantly alter the magnitude and the frequency content of force variability [23].

**Fig 1:**
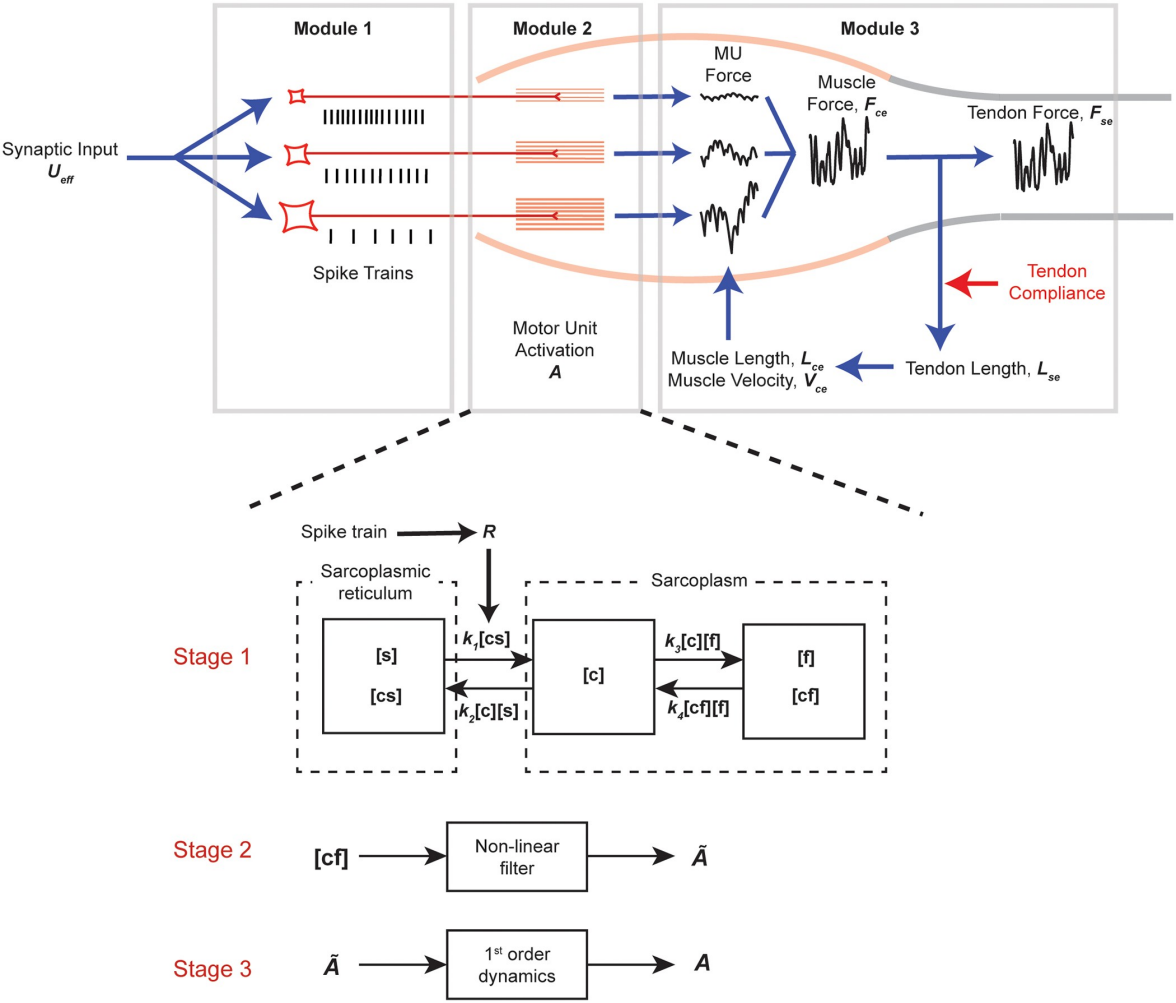
Schematic representation of our new model of a motor unit pool. The model consists of three modules. Module 1 converts synaptic input, *U*_*eff*_, into spike trains of individual motor units. Module 2 turns spike trains into motor unit activation, *A*, through three-stage process shown below. Stage 1 simulates calcium kinetics driven by action potentials (*R*). The calcium kinetics is described using five states, [*s*], [*cs*], [*c*], [*f*] and [*cf*] with associated rate constants (*k*_1_, *k*_2_, *k*_3_ and *k*_4_) between those states. Stage 2 converts [*cf*] into the intermediate activation, $\tilde{A}$, through a non-linear filter, which describes cooperativity and saturation of calcium binding and cross-bridge formation. Stage 3 introduces an additional first-order dynamics to generate motor unit activation, *A*, from $\tilde{A}$. Module 3 describes the contraction dynamics between muscle and a series elastic element and generates tendon force, *F*_*se*_, as the output. The detail descriptions of each module are given in the text.

To address these issues and re-evaluate the contribution of motor unit properties to force variability, we have developed a new model drawn schematically in Fig 1. This model was developed based on the architectural and physiological properties of the human *tibialis anterior* muscle as described below. To do so, we performed thorough analyses of extensive experimental data on which our model is based. We attempt to justify and construct many details of this model according to underlying physiological mechanisms, some of which turn out not critical for our general conclusion. By pointing to our sources, we believe our paper will be useful for anyone attempting to adapt or extrapolate our model for other purposes.

#### Motor unit architecture

The model of the *tibialis anterior* muscle consists of 200 motor units (*N* = 200). The number of motor units was estimated by subtracting the number of Ia and Ib afferent nerves from that of large-diameter nerve fibers reported in the human *tibialis anterior* muscle. Feinstein et al. [50] reported 742 large-diameter nerve fibers associated with the *tibialis anterior* muscle. We assumed 284 of those are Ia afferent nerves given the reported number of muscle spindles in the *tibialis anterior* muscle [51, 52]. We further assumed that the ratio for Golgi tendon organs to muscle spindles is ∼0.9 (in the upper range of ratios for various muscles reported by Jami et al. [53]), resulting in 258 of the remaining nerve fibers belonging to Ib afferent nerves. It is important to note that the number of motor units estimated in this method (i.e. 200) is much smaller than the estimated number (i.e. 445) by Feinstein et al. [50], where the author assumed 60% of the large nerve fibers to be motoneurons. Our model value is slightly lower than the number of *tibialis anterior* motor units estimated from single motor unit recordings by McComans [54].

#### Module 1: Conversion of synaptic input into discharge patterns

The motor unit pool is driven by an effective synaptic input, *U*_*eff*_, whose normalized value ranges from 0 and 1. The time course of the effective synaptic input was applied equally to all motor units in a pool. It is important to note that some synaptic inputs (e.g. Ia afferent and rubrospinal inputs) may be distributed non-uniformly across motor units [55, 56] and such non-uniform distribution can influence the range of recruitment thresholds and the general frequency-input relationship of a motor unit pool [57, 58]. However, it is difficult to accurately simulate how a non-uniform distribution of synaptic input would affect those parameters due to limited experimental data from humans and potential anatomical differences across muscles and across species (e.g. potential absence of the rubrospainal tract in humans [59]). Instead, the range of recruitment thresholds and the frequency-input relationship were directly manipulated assuming the uniform distribution of synaptic inputs across motor units.

*Peak and minimal discharge rate*: Intracellular recordings of cat motoneurons have shown that the frequency-current relationship of a motoneuron (discharge rate vs. injected current to the motoneuron soma) is best described as two linear ranges: primary and secondary [10, 60–62]. Almost all motoneurons can sustain repetitive discharges in the primary range [62, 63] and many do not show the secondary range [61, 62, 64]. Motor unit forces reach 65-95% of their respective peak tetanic force at the transition frequency to the secondary range [57]. Furthermore, motor unit forces are approximately 10% of their respective peak tetanic force when motoneurons initiate their repetitive discharges [56, 65].

**Fig 2:**
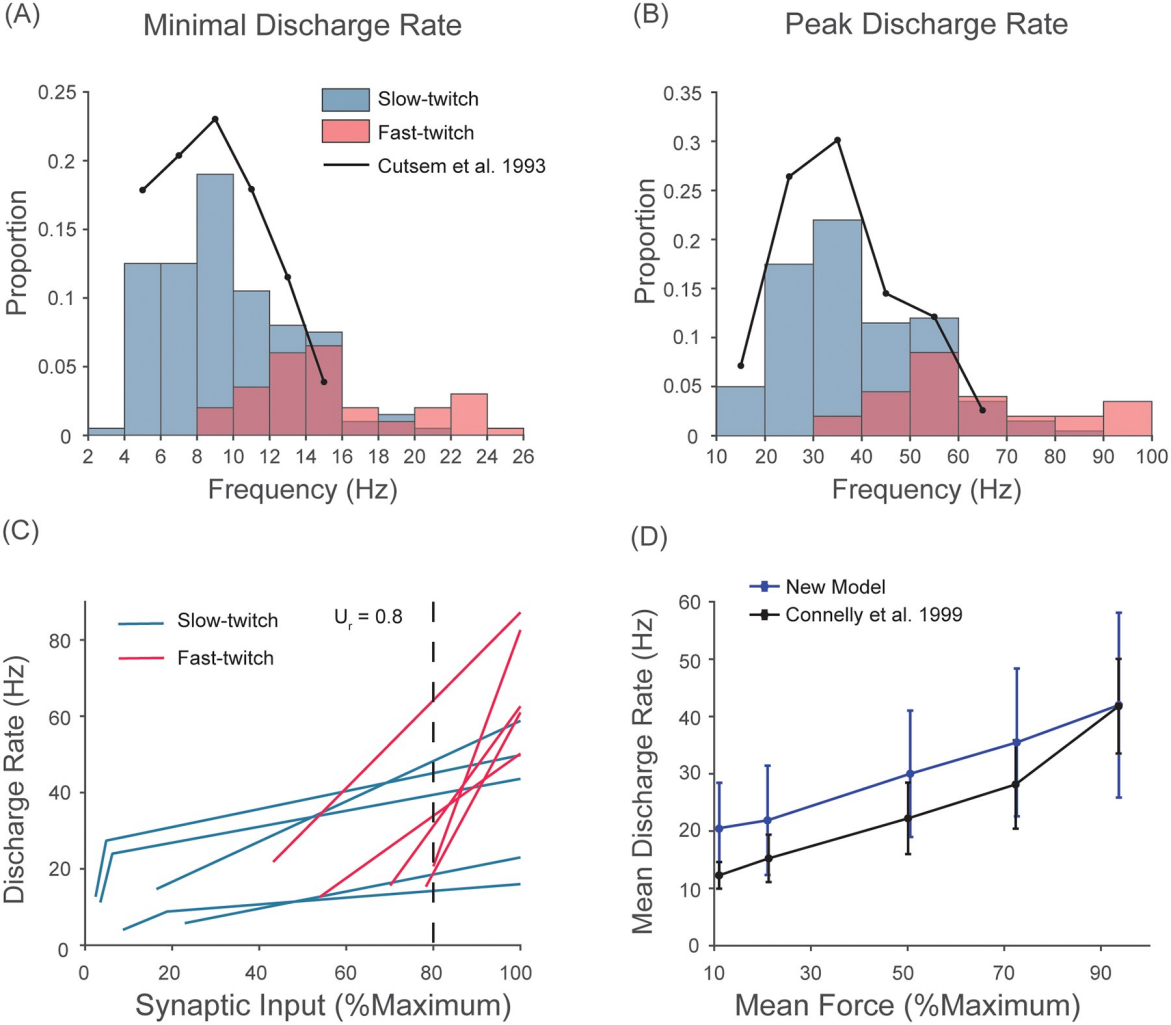
New recruitment scheme of our new model mimics discharge patterns of human *tibialis anterior* motor units. A) The distribution of minimal discharge rates of all motor units compared against experimental data from Cutsem et al. [46]. B) The distribution of peak discharge rates of all motor units compared against experimental data from Cutsem et al. [46]. C) The frequency-synaptic input relationship of the selected motor units (n = 10). U_r indicates the level of synaptic input at which all motor units are recruited. Lower-threshold motor units (below 10%of the maximal synaptic input) show rapid acceleration upon recruitment and saturation of their discharge rates. Higher-threshold units (red) linearly increase their discharge rates up to the maximal synaptic input. D) Mean discharge rates of all active units at four different force levels (11, 21, 50, 72 and 93 % of the maximum force)compared against experimental data from Connelly et al. [68].

We assume in our model that the discharge rate of individual motor units is modulated between their minimal discharge rate (*MDR*_*i*_) and peak discharge rate (*PDR*_*i*_), which were determined based on *f*_0.5*i*_, the discharge rate at which the motor unit activation reaches 50% of the maximum determined empirically (see below). As done previously by Song et al. [66], *PDR*_*i*_ and *MDR*_*i*_ were set equal to 2 ⋅ *f*_0.5*i*_ and 0.5 ⋅ *f*_0.5*i*_, respectively. The mean levels of motor unit activation are approximately 16% and 85% at their minimal and peak discharge rates, respectively, consistent with the experimental observations discussed above. The resulting distributions of *MDR* and *PDR* resemble those obtained from the human *tibialis anterior* muscle by [46] (Fig 2A and 2B). It is important to note that the value of *f*_0.5*i*_ for individual motor units is closely linked to the speed of muscle fiber contraction (i.e. contraction time) and as a result we explicitly assume that the discharge rates of individual motor units and their mechanical properties are closely matched. We assumed this relationship based on the following empirical evidence in animals provided by Kernell and others and later confirmed by MacDonell et al. [67] in humans.

Motor units with faster calcium kinetics and cross-bridge cycling (i.e. faster contraction time) require faster discharge rates to achieve the same relative force with respect to their peak tetanic force [69, 70]. Consistently, the peak discharge rate of motor units achieved during the maximal voluntary contraction is inversely correlated with its contraction time [71, 72]. Furthermore, motoneurons that innervate fast-contracting muscle fibers tend to initiate their repetitive discharge at faster discharge rates [73] and can reach higher discharge rates compared to those that innervate slow-contracting muscle fibers [74–76]. The duration of afterhyperpolarization, which is one of the main mechanisms that regulate the minimal discharge rate of a motoneuron [56, 73], is correlated with twitch contraction time [77–81]. Also, the range in which motoneurons initiate and maintain repetitive discharge patterns (primary range) correspond well with the range in which modulation of force is the greatest [62, 72, 75] (i.e. the range between 2 ⋅ *f*_0.5*i*_ and 0.5 ⋅ *f*_0.5*i*_). This ‘motoneuron-muscle unit speed-match’ [75] is likely energetically efficient [82–84] and appropriate for different functions motor units with different contraction speed serve [85]. Furthermore, this speed-match is regulated by both genetic and epigenetic factors [86–89]. This matching starts to occur during the embryonic development [89]. Studies on cross-innervation of motor units (e.g. surgical innervation of motoneurons from slow-twitch units to muscle fibers from fast-twitch units) show that epigenetic factors further reinforce the ‘motoneuron-muscle unit speed-match’ formed in early stages of development by demonstrating orthograde (motoneurons) and retrograde (muscle fibers) adaptations within motor units [86, 88].

The motoneuron-muscle unit speed-match has been observed, albeit indirectly, in humans. MacDonell et al. [67] showed that motor units with longer afterhyperpolarization tend to have the lower minimal discharge rates and slower contraction times, suggesting that motor units with the lower minimal discharge rates tend to have slower contraction times. Furthermore, the peak discharge rate of *soleus* motor units (almost exclusively slow-twitch units, i.e. slow contraction times) are significantly slower than that of biceps motor units whose contraction times are much faster than *soleus* motor units [71]. Furthermore, reductions in contraction time of *tibialis anterior* motor units with aging is accompanied by reductions in their discharge rates at the same relative force levels with respect to their maximal voluntary contraction [68].

*Recruitment threshold*: The distribution of recruitment thresholds of motoneurons in a motor unit pool is determined by intrinsic electrical properties of motoneurons (e.g. input resistance and rheobase) as well as the organization of excitatory, inhibitory and neuromodulatory inputs across the constituent motor units [55, 56, 90–92]. The distribution of rheobase (an index of excitability of motoneurons [92]) across motoneurons in a pool is skewed to the high rheobase [92–94]. Similarly, it was found both in human and animal that the distribution of recruitment thresholds in a pool as a fraction of the maximal force output follows an exponential distribution where a larger proportion of units are recruited at low force levels (i.e. weaker synaptic inputs) [95–98]. As such, we modeled the distribution of recruitment thresholds by fitting an exponential function (*fit* function in MATLAB) that spans the range from the lowest recruitment threshold (*U*_1_) to the highest recruitment threshold (*U*_*r*_) in the unit of effective synaptic drive (0-1). The value of *U*_1_ was always set to 0.01 (1% of the maximal synaptic input). The value of *U*_*r*_ was set to 0.8 by default (i.e. all motor units are recruited at 80% of the maximal synaptic input) based on experimental findings in the human *anterior tibilais* muscle [46, 47].

The order in which individual motor units are recruited follows Henneman’s size principle where smaller motor units are always recruited before larger ones [92, 93, 95–114]. This recruitment order tends to be robust regardless of types of synaptic inputs in most muscles [102, 103, 105] with the possible exception of certain hand muscles [115, 116]. Accordingly, we assigned lower recruitment thresholds to motor units with smaller peak tetanic force. This positive correlation between the recruitment threshold and peak tetanic force (therefore twitch force) is consistent with previous experimental findings in humans [97, 98, 100, 115]. The methods we used to determine the peak tetanic force of individual motor units are described below.

*Recruitment scheme*: We developed a new recruitment scheme, which resembles experimentally observed motor unit discharge patterns in humans (i.e. the rate limiting of low-threshold motor units [37, 57, 58, 63, 90, 109, 117–121] presumably due to motoneuron intrinsic mechanisms such as adaptation [75, 122–126] and persistent-inward current [56, 119, 127–130]). We did so by modifying and combining previously proposed methods [30, 66, 131]. Specifically, this new scheme demonstrates low-threshold motor units whose recruitment threshold is below 10% of the maximal synaptic input show rapid acceleration and saturation of their discharge rates, while the remaining ‘higher-threshold’ units linearly increase their discharge rates and reach their peak discharge rates at the maximal synaptic input (Fig 2C). The frequency-input relationship of the high-threshold units is modeled using the following equation:

$$DR_{i}\left(t\right)=g_{ei}\cdot \left[U_{eff}\left(t\right)-RT_{i}\right]+MDR_{i}$$ (2)

$$\begin{array}{ccc} & g_{ei}=\frac{PDR_{i}-MDRi}{1-RT_{i}}, & \end{array}$$ (3)

where *RT*_*i*_ is recruitment threshold of the *i*-th motor unit in a unit of *U*_*eff*_ (0-1), and *MDR*_*i*_ and *PDR*_*i*_ are the minimal and peak discharge rates of that motor unit, respectively. Note that the gain of the frequency-input relationship, *g*_*e*_, differs across motor units depending on the values of *RT*_*i*_, *MDR*_*i*_ and *PDR*_*i*_.

The frequency-input relationship of the low-threshold units is described as two linear functions using the following equations:

$$DR_{i}\left(t\right)=\{\begin{array}{cc} \lambda_{i}\cdot k_{ei}\cdot \left[U_{eff}\left(t\right)-RT_{i}\right]+MDR_{i} & RT_{i}<=U_{eff}\left(t\right)<=U_{ti}\\ PDR_{i}-k_{ei}\cdot \left[1-U_{eff}\left(t\right)\right] & U_{eff}\left(t\right)>U_{ti} \end{array}$$ (4)

$$\begin{array}{cc} & \lambda_{i}=-100RT_{i}+21 \end{array}$$ (5)

$$\begin{array}{cc} & k_{ei}=\frac{f_{t}\cdot f_{0.5i}-MDR_{i}+\lambda_{i}\cdot \left(PDR_{i}-f_{t}\cdot f_{0.5i}\right)}{\lambda_{i}\cdot \left(1-RT_{i}\right)} \end{array}$$ (6)

$$\begin{array}{ccc} & U_{ti}=\frac{k_{ei}-\left[PDR_{i}-\left(f_{t}\cdot f_{0.5i}\right)\right]}{k_{ei}}. & \end{array}$$ (7)

These equations were derived such that the slope of the first linear region (i.e. when *U*_*eff*_ < *U*_*ti*_) is much steeper than that of the second linear region, where *U*_*ti*_ determines the level of synaptic input at which a given motor unit transitions from the first linear region into the second. λ_*i*_ determines the relative slope of the first to second linear function, which was modeled to decrease from a value of 30 to 1 linearly with *RT*_*i*_. Given the value of λ_*i*_, the value of *k*_*ei*_ is then calculated, which determines the rate at which the discharge rate increases with a given increment in synaptic input in the second linear function. The transition frequency, *f*_*t*_, describes the frequency at which the slope transitions from the first to the second linear function. The value of *f*_*t*_ was chosen to be 1.1 in the unit of *f*_0.5_, the discharge rate required to reach half the peak tetanic activation of a motor unit. The value of 1.1 ⋅ *f*_0.5_ corresponds, on average, to 57.2% (SD = 0.2) of the maximal motor unit activation and achieves 75.1% (SD = 7.5) of fusion at optimal muscle length (i.e. 1.0*L*_*ce*_), which are consistent with motor unit force achieved at saturation discharge rates of low-threshold motor units observed in human by Fuglevand et al. [119]. All of these parameters were set to qualitatively mimic discharge patterns of human motor units (e.g. Fig 2 in [109] and Fig 5 in [63]) as shown in Fig 2C.

The resulting discharge patterns of motor units are compatible with experimental observations from human *tibialis anterior* motor units when we approximate the contraction time of individual motor units to experimentally reported values (see the section on contraction time below). Fig 2D shows that the pattern of changes in mean discharge rates of all active motor units in our model at four different force levels (11, 21, 50, 72 and 93% of the maximal force) match well to experimental data from Connelly et al. [68]. Note that this continuous increase in mean discharge rate cannot be replicated when we assumed the conventional onion-skin recruitment scheme where the peak discharge rates of motor units decrease with their size. Higher discharge rates in our model compared to experimental data by Connelly et al. [68] arise from higher *f*_0.5*i*_ in our model as indicated in Fig 2A and 2B) (note again that the minimal and peak discharge rates depend on *f*_0.5*i*_). This difference is not likely due to the way in which we define the minimal and peak discharge rates, but rather because our model requires higher discharge rates to attain 50% of the maximal activation for a given contraction time as indicated by the lower mean ratio of inter-spike interval at *f*_0.5_ to contraction time (1.18) compared to experimentally reported value of 1.29-1.34 in human muscles [132]. In other words, the inter-spike interval at *f*_0.5*i*_ in our model for a given contraction time is shorter (i.e. higher discharge rate).

*Variability in motoneuron discharge*: Variability in timing of motoneuron discharge was introduced using the method described by Fuglevand et al. [30]. The *j*-th spike time of the *i*-th motor unit is described in the following equation:

$$\begin{array}{cc} t_{i,j}=\mu +\mu \cdot cv\cdot Z+t_{i,j-1}. & \end{array}$$ (8)

The mean inter-spike interval, *μ*, is calculated from discharge rate predicted from the Eqs 2–4. The value of *cv* determines the degree of stochasticity in motor unit discharges as per coefficient of variation (CoV) for inter-spike intervals (ISIs). For each discharge, the value of Z was randomly drawn from the standard normal distribution whose values range from -3.9 to 3.9 [30], which simulates a renewal process where inter-spike intervals between successive spikes are uncorrelated and the distribution of inter-spike intervals follow a Gaussian distribution.

The value of *cv* was set constant in a set of simulations where CoV of ISIs were varied from 0 to 20% to quantify how the degree of stochasticity influence the overall amplitude of force variability. Otherwise, the value of *cv* was made to depend on the value of *U*_*eff*_ as described in Moritz et al. [37] to compare the predicted motor noise from our model to the experimentally measured amplitude of force variability reported by Tracy [133]. We did so using the equation provided by Moritz et al. [37], which was modified as follows:

$$\begin{array}{cc} cv=10+20e^{-(U_{eff}-RT_{i})/2.5}. & \end{array}$$ (9)

This equation simulates an exponential decline in CoV of ISIs with increasing force levels observed experimentally by Moritz et al. [37] and supported theoretically as shown in S1 Appendix.

#### Module 2: Conversion of spike trains into motor unit activation

Module 2 converts motor unit spike trains into motor unit activation (a value between 0 and 1), *A*, via three-stage processes (Fig 1). First, a motor unit spike train is converted into a state variable, [*cf*], a fraction of cross-bridges bound to calcium that can participate in force generation (*Stage 1*). Second, the state variable, [*cf*], is then passed through a non-linear filter that accounts for cooperativity and saturation of calcium binding and cross-bridge formation (*Stage 2*). Third, the output of the non-linear filter, $\tilde{A}$, is converted into motor unit activation, *A*, using the first-order dynamics (*Stage 3*). The majority of Module 2 was taken from the previous model from cat *soleus* muscle by Kim et al. [134] and simplified as described below. This simplification allowed us to replicate the activation-frequency relationship and fusion of motor unit twitches recorded experimentally with a fewer, more tractable, number of parameters for individual motor units compared to that in [134] (see Results).

*Stage 1*: We used the simplified model of calcium kinetics originally proposed by Westerblad and Allen [135] and later adapted by Kim et al. [134] for cat *soleus* muscle. We simplified the model by removing the interaction between free calcium ions and calsequestrin within the sarcoplasmic reticulum and the interaction between free calcium ions and calcium buffering proteins in the sarcoplasm. These simplifications allowed us to describe calcium kinetics with only five states: the concentrations of bound and unbound sarcoplasmic reticulum calcium-binding sites, [*cs*] and [*s*], that of free calcium ion, [*c*], those of bound and unbound myofilament calcium-binding sites, [*f*] and [*cf*] with associated rate constants (*k*_1_, *k*_2_, *k*_3_ and *k*_4_) between those states (Stage 1 in Fig 1). Assuming the ratio of the total number of calcium, *C*, and sarcoplasmic reticulum binding sites, *S*, to the total number of myofilament binding sites, *F*, the dynamics of free calcium, [*c*], and that of calcium bound to myofilaments, [*cf*], can be expressed using the following equations [136]:

$$\begin{array}{cc} \frac{d[c]}{dt}=k_{1}\left(C-\left[c\right]-\left[cf\right]\right)R-k_{2}\left[c\right]\left\{S-C+\left[c\right]+\left[cf\right]\right\}-\left(k_{3}\left[c\right]-k_{4}\left[cf\right]\right)\left(1-\left[cf\right]\right) & \end{array}$$ (10)

$$\begin{array}{cc} \frac{d[cf]}{dt}=\left(1-\left[cf\right]\right)\left(k_{3}\left[c\right]-k_{4}\left[cf\right]\right). & \end{array}$$ (11)

$$\begin{array}{cc} R=\sum_{i=1}^{n}(1-exp(-\frac{t-t_{i}}{\tau_{1}}))\cdot exp(-\frac{t-t_{i}}{\tau_{2}}) & \end{array}$$ (12)

$$\begin{array}{cc} k_{4}=\frac{k_{4i}}{\left(1+\gamma \cdot A\right)}. & \end{array}$$ (13)

*k*_4*i*_ is the initial rate constant for *k*_4_. As done previously in Kim et al. [134], the value of *k*_4_ is made to depend on the motor unit activation, *A*, to simulate the cooperativity of cross-bridge formation [135]. The value of *γ* in Eq 13 was set to 0.8 at *L*_0_. Eq 12 drives calcium diffusion into sarcoplasm upon arrival of action potentials as in Kim et al. [134].

The parameters, *C*, *S* and *F* have the following relationships:

$$\begin{array}{cc} S=\alpha_{S}C & \end{array}$$ (14)

$$\begin{array}{cc} C=\alpha_{C}F, & \end{array}$$ (15)

where the value of *F* was assumed to be 1. The value of *α*_*C*_ was set constant to 1.8 across individual motor units, which is slightly higher than the ratio of the total free calcium ion concentration (1.5 *mM*) to the total concentration of sarcoplasm binding sites (1.0 *mM*) used in Kim et al. [134] and Westerblad and Allen [135]. The value of *α*_*S*_ was varied around that used in Kim et al. [134] and Westerblad and Allen [135] (cf. the concentration of sarcoplasmic reticulum calcium bindings sites of 30 *mM*) across motor units as described below.

*Stage 2*: We further introduce cooperativity and saturation of calcium binding and cross-bridge formation using the Hill equation [83, 137] described below:

$$\begin{array}{cc} \tilde{A}=\frac{{\left(\left[cf\right]\cdot S_{i}\cdot Y\right)}^{N}}{{\left(\left[cf\right]\cdot S_{i}\cdot Y_{i}\right)}^{N}+K^{N}}. & \end{array}$$ (16)

The coefficients, *N* and *K*, describe non-linearity due to cooperativity and saturation of calcium binding and cross-bridge formation, which are determined for each motor unit. The parameters, *S*_*i*_ and *Y*, describe the sag and yield properties of slow and fast-motor units [138, 139] using the following equations:

$$\dot{S_{i}}\left(t\right)=\frac{a_{S}-S_{i}\left(t\right)}{T_{S}},$$ (17)

$$\begin{array}{ccc} & a_{S}=\{\begin{array}{c} a_{S1},DR_{i}/f_{0.5i}<0.1\\ a_{S2},DR_{i}/f_{0.5i}\geq 0.1 \end{array} & \end{array}$$ (18)

$$\begin{array}{cc} \dot{Y_{i}}\left(t\right)=\frac{1-c_{Y}\left[1-exp\left(-\frac{|V_{ce}|}{V_{Y}}\right)\right]-Y\left(t\right)}{T_{Y}}. & \end{array}$$ (19)

All parameters associated with Eqs 17&19 are same as ones given in Song et al. [66] except *a*_*S*1_ and *T*_*S*_, which were set to 20 and 15 ms to replicate similar behavior of sag.

*Stage 3*: The intermediate activation, $\tilde{A}$, was then converted into motor unit activation, *A*, as was done in Kim et al. [134], using the following first-order dynamics:

$$\begin{array}{cc} \dot{A}=\frac{\tilde{A}-A}{\tau_{3}}. & \end{array}$$ (20)

The dependence of *τ*_3_ on [*cf*] included in the previous model by Kim et al. [134] was removed as it did not seem to affect our results significantly.

*Length-dependence of activation-frequency relationship*: The activation-frequency relationship is known to depend on muscle length [138]. Therefore, we introduced length-dependence on four of the 12 free parameters, *k*_3_, *N*, *K*, and *γ* using the following relationship as a function of muscle length, *L*_*ce*_, [134, 136]:

$$\begin{array}{cc} x=\phi_{i}\cdot x_{0}\cdot \left(1-L_{ce}\right)+x_{0}, & \end{array}$$ (21)

where *x*_0_ the value of a given parameter at *L*_0_ and *x* is their predicted value at muscle length ≠ *L*_0_. *ϕ*_*i*_ is the coefficient to be determined for each motor unit. The same value of *ϕ*_*i*_ was used for all four parameters for a given motor unit. *k*_3_ was chosen as done previously by Kim et al. [134]. We added length-dependence on three additional parameters, *N*, *K*, and *γ*, to better replicate the experimental data provided by Brown et al. [138].

*Determining free parameters*: Through Stage 1-3, various properties of individual motor units (e.g. contraction time, activation-frequency relationship, etc.) at the optimal muscle length (i.e. *L*_0_) were modeled by adjusting 12 free parameters (*α*_*S*_, *α*_*C*_, *k*_1_, *k*_2_, *k*_3_, *k*_4_, *γ*, *N*, *K*, *τ*_1_, *τ*_2_ and *τ*_3_) in the equations described above. We did so in the following manner: (1) Seed parameter sets were manually constructed such that each parameter set can generate a twitch response with contraction time within the specified range (see the section on *contraction time* below) and replicate the activation-frequency relationship similar to that modeled previously by Brown et al. [138]. (2) The values of *α*_*S*_, *k*_1_, *k*_2_, *k*_3_, *k*_4_, *K* and *τ*_1_ in the seed set were then randomly perturbed from their original values to generate various values of contraction time and twitch-tetanus ratio (see the section on twitch-tetanus ratio below) while we systematically decreased the values of *τ*_2_ and *τ*_3_ from slow to fast motor units. We kept the values of *γ* and *N* constant. Once we produced a set of parameters in this manner, we manually assigned a set of parameters to each motor unit such that we could replicate the relationship between contraction time and twitch-tetanus ratio as described below.

The value of *ϕ*_*i*_ for each motor unit was fitted to minimize an error between the predicted activation-frequency relationships at four different muscle lengths (0.8*L*_0_, 0.9*L*_0_, 1.1*L*_0_ and 1.2*L*_0_) in our model and those given in [138]. We fitted *ϕ*_*i*_ separately for muscle lengths shorter than *L*_0_ and longer than *L*_0_ as done previously in Kim et al. [134].

#### Module 3: Musculotendon dynamics

To simulate the contraction dynamics of a musculotendon unit during an externally isometric contraction (musculotendon unit length = constant), we used the model of a musculotendon unit proposed previously [66, 138, 140–142]. This model comprises of a mass, a contractile element, two passive parallel elements and a series-elastic element [66]. The contraction dynamics of this system is described as follows [143]:

$$A_{ce}\cdot L_{ce0}=\frac{1}{M_{m}}\left[F_{0}\cdot F_{se}\cdot cos\alpha -\left(F_{ce}+F_{0}\cdot F_{pe1}\right)\cdot cos^{2}\alpha \right]$$ (22)

$$\begin{array}{cc} & +\frac{{\left(V_{ce}\cdot L_{ce0}\right)}^{2}\cdot tan^{2}\alpha }{L_{ce}\cdot L_{ce0}} \end{array}$$ (23)

$$\begin{array}{ccc} & L_{mt}=L_{ce}\cdot L_{ce0}\cdot cos\alpha +L_{se}\cdot L_{se0}=constant & \end{array}$$ (24)

where *M*_*m*_, *α*, *L*_*ce*_, *V*_*ce*_, *A*_*ce*_, *L*_*se*_ and *L*_*mt*_ are muscle mass, pennation angle, muscle length normalized to optimal muscle length, normalized muscle velocity, normalized muscle acceleration, tendon length normalized to optimal tendon length and musculotendon unit length in cm. *F*_0_ determines maximal tetanic force of muscle as described in the next section. The value of these parameters are listed in Table 1. *F*_*ce*_, *F*_*pe*1_ and *F*_*se*_ are force from the contractile element, passive element 1, and series elastic element, respectively. *F*_*ce*_ is computed as follows:

$$\begin{array}{cc} F_{ce}=\sum_{i=1}^{n}PT_{i}\cdot A_{i}\cdot \left(FL_{i}\cdot FV_{i}+F_{pe2}\right). & \end{array}$$ (25)

*PT*_*i*_ is peak tetanic force of individual motor units. *F*_*pe*2_ is passive, resistive force against shortening. *FL*_*i*_ and *FV*_*i*_ are force-length and force-velocity properties of individual motor unit as described as follows:

$$\begin{array}{cc} FL_{i}=exp\left(-{|\frac{L_{ce}^{\beta }-1}{\omega }|}^{\rho }\right). & \end{array}$$ (26)

$$\begin{array}{cc} FV_{i}=\{\begin{array}{c} \left(V_{max}-V_{ce}/\left[V_{max}+\left(c_{v0}+c_{v1}*L_{ce}\right)V_{ce}\right]\right),V_{ce}\leq 0\\ {}[b_{v}-\left(a_{v0}+a_{v1}L_{ce}+a_{v2}L_{ce}^{2}\right)V_{ce}]/\left(b_{v}+V_{ce}\right),V_{ce}<0 \end{array}. & \end{array}$$ (27)

The values of the coefficients in the above equations depend on fiber types and can be found in Table 1. in Song et al. [142].

**Table 1:** Model parameters for muscle based on architectural parameters of *tibialis anterior* muscle [144, 145].

Muscle mass, *M*_*m*_ (g)	150
Optimal muscle length, *L*_*ce*0_ (cm)	6.8
Optimal tendon length, *L*_*se*0_ (cm)	27.5
Pennation angle, *α* (deg)	9.6

*Muscle maximal tetanic force*: We determined the maximal tetanic force of muscle (*F*_0_) using the following equation given in [146]:

$$\begin{array}{cc} F_{0}=\frac{M_{m}\cdot cos\left(\alpha \right)\cdot \epsilon }{\rho \cdot L_{ce0}}, & \end{array}$$ (28)

where *M*_*m*_, *ρ*, *ϵ* and *L*_*ce*0_ are muscle mass, muscle density (1.06 g/cm^3^), specific tension (31.8 N/cm^2^) and optimal fascicle length, respectively.

**Fig 3:**
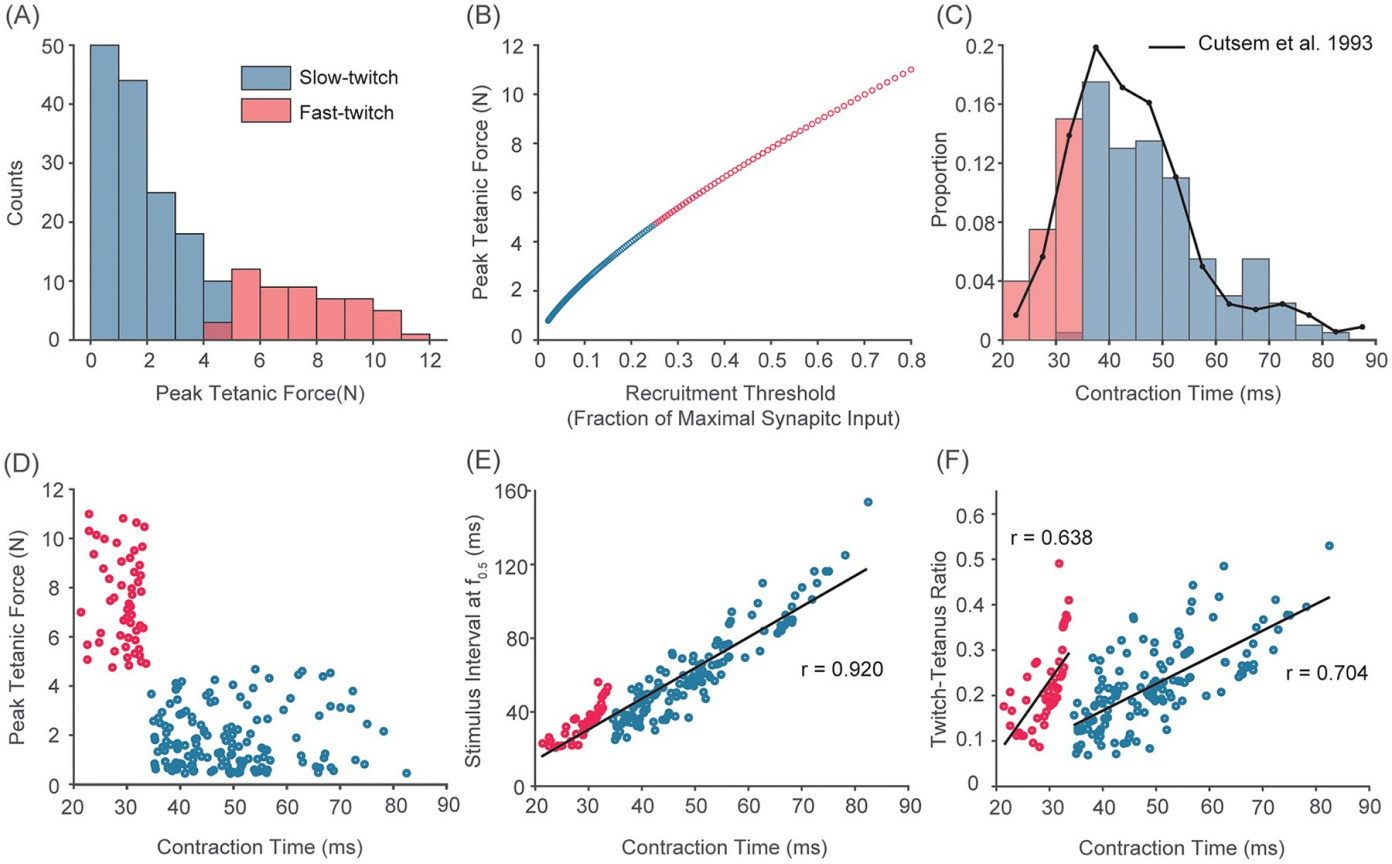
Population characteristics of our default motor unit pool. A) Histogram of peak tetanic force of 200 motor units (blue: slow-twitch, red: fast-twitch). The distribution follows an exponential distribution where a large portion of motor units produce relatively smaller tetanic force. B) Peak tetanic forces as a function of recruitment thresholds. The relationship assumes the size-principle (e.g. Henneman, 1957): smaller units get recruited earlier than larger units. C) Histogram of contraction time of 200 motor units. The distribution follows the Rayleigh distribution which spans 20 ms to 85 ms, which closely matched the distribution observed in human *tibialis anterior* muscle by Cutsem et al. (1993). D) The relationship between peak tetanic force and contraction time. Slow-twitch units (in blue) have slower contraction time and smaller peak tetanic force. Within each fiber type, no correlation between peak tetanic force and contraction time was assumed. E) The relationship between contraction time and stimulus interval at the frequency at which half the tetanic force is achieved *f*_0.5_. Consistent with previous experimental data [69, 70], those parameters in our default motor unit pool are highly correlated (*r* = 0.920). F) The relationship between contraction time and twitch-tetanus ratio. Within each fiber type, twitch-tetanus ratio is positively correlated with contraction time (correlation coefficients, *r*, are 0.638 and 0.704 for slow-twitch and fast-twitch units, respectively).

*Motor unit tetanic force*: Maximal tetanic force of individual motor units, *PT*_*i*_, was determined in using the following equation:

$$\begin{array}{c} PT_{i}=F_{0}\frac{exp(b\cdot i)}{\sum_{n=1}^{N}exp\left(b\cdot i\right)} \end{array}$$ (29)

$$\begin{array}{cc} b=log(RP)/N & \end{array}$$ (30)

where *i*, *N* and *RP* are an index of motor units, the number of motor units in a pool and the range of peak tetanic force defined in the following section. This generates an exponential distribution of peak tetanic force (Fig 3A) such that a greater proportion of motor units produce smaller peak tetanic force [147, 148]. This also results in an exponential distribution of twitch force as documented extensively in previous studies in both cat [104, 149, 150] and humans [46, 97, 109, 151]. Furthermore, peak tetanic force of motor units is positively correlated with their recruitment thresholds (Fig 3B) according to Henneman’s size principle as described above.

*Range of maximal tetanic force of motor units*: As mentioned above, the Fuglevand model used a 100-fold range in the peak *twitch* force between the smallest and largest motor units. The rational for this relatively large range was based on previous empirical observations [49, 97, 98, 104, 109, 147, 149, 150, 152–156]. However, there are methodological limitations with the use of twitch amplitude to infer the size of motor units as described by Kernell [65] and it is likely that these empirical observations might have underestimated peak twitch/tetanic forces of smaller motor units for the following reason. Heckman et al. [157] found that prior or concurrent movement potentiated tetanic force of motor units whose amplitude was less than 200 mN (ca. 20 g). This effect of movement potentiation was largest in smaller motor units. Their proposed mechanism for such an effect was that movement reduced the opposing force of thixotrophic and viscoelastic connective tissues around muscle fibers (i.e. endomysium). Importantly, this was considered not due to mechanical properties of muscle fibers per se. This raises an important issue measuring twitch/tetanic force from an intact muscle (as opposed to skinned-fiber preparations) because it suggests the smallest twitch/tetanic force, where this effect was largest, might have been larger than actually recorded. Consistent with this view, Olson and colleagues reported peak tetanic forces of 108 motor units of flexor digitrum longus from three cats averaged to 9.6 g, which was lower than average peak tetanic force of 14.5 g estimated from the number of motor units and peak tetanic force of the muscles [147].

Instead, maximal tetanic force of individual motor units is determined by three factors: 1) innervation ratio, 2) mean cross-sectional area of muscle fibers, and 3) specific tension [49, 153, 158] as described in the following equation:

$$\begin{array}{c} f_{i}=N\cdot A\cdot \epsilon_{i} \end{array}$$ (31)

where *f*_*i*_, *N*, *A* and *ϵ*_*i*_ are tetanic force of *i*-th motor unit, the number of muscle fibers, mean cross-sectional area of muscle fibers and specific tension of *i*-th motor unit, respectively. In fact, these three factors can explain 98.2-99.5% of variance in peak tetanic force across motor units of different fiber types [153]. Then, we can approximate a possible range of *f*_*i*_ if we know the possible ranges of *N*, *A* and *ϵ*_*i*_. It is important to note that the glycogen depletion method used to estimate these measures might be prone to error, especially for fatigue-resistant units [158]. However, insufficient depletion of glycogen for fatigue-resistant units is expected to underestimate innervation ratio and therefore peak tetanic force of smaller motor units [159]. Therefore, one would expect the range of peak tetanic force to be smaller, not larger than reported previously.

The difference in mean cross-sectional area between the smallest and largest units ranges from 1.7 to 7 fold [49, 153, 160–162]. The difference in innervation ratio ranges from approximately 2 to 8 fold [49, 153, 160, 161]. Specific tension, on the other hand, does not seem to differ across motor unit types based on observations from skinned muscle fiber preparation [163] and intact muscle fibers [160, 161], although some studies have found a lower value of specific tension for slow motor units [153, 158, 160]. This discrepancy in findings may be explained by the above mentioned observation by Heckman et al. [157] that peak tetanic force of smaller motor units might have been underestimated due to the opposing force of connective tissues because specific tension is derived from the measured peak tetanic tension and cross-sectional area. Finally, Kanda and Hashizume [153] reported 20-fold difference in total cross-sectional area in rat medial gastrocnemius muscle (muscle with mixed fiber-type). We chose 25-fold difference as a default value in our simulation, slightly larger than the range reported by Kanda and Hashizume [153] considering potential under-sampling of the entire population.

*Assigning fiber types to each motor unit*: Although some motor units properties such as peak tetanic force and contraction time can be modeled continuous across motor units as done previously [30], other properties can differ in a discrete manner across fiber types such as sag and yield as modeled in Eqs 17 and 19. For simplicity, we divide motor units into slow-twitch (type S) and fast-twitch (type F) types as in other models [66, 142]. There exists strong empirical evidence that fast-twitch (type F) units generate higher unit force than slow-twitch (type S) units [49, 114, 153, 160, 161, 164]. Thus, we assumed that there is no overlap in peak tetanic tension between slow and fast-twitch units for simplicity. We then divided the motor unit pool into two fiber types such that 40% of muscle maximal force arises from slow-twitch units and the rest from fast-twitch units, which results in 147 units out of 200 being slow-twitch.

*Contraction time*: Contraction time is defined as the time a motor unit twitch response takes to reach its peak amplitude from the baseline force level. This parameter reflects the speed of calcium kinetics and cross-bridge cycling, which determines the speed at which a given motor unit can generate force. Contraction time has been used as an important indicator to differentiate slow-twitch and fast-twitch units [164]. Contraction time of fast and slow-twitch units does not overlap [164]. Based on previous experimental observations [97, 104, 147–150, 154, 159, 165–169], we modeled the distribution of contraction time to follow a Rayleigh distribution. We set the distribution to span 25 ms to 90 ms (Fig 3C) to be consistent with experimental data on human *tibialis anterior* muscle [46]. We assigned the 147 slow-twitch motor units to slower contraction times with no overlap with fast-twitch motor units (53 units) (Fig 3D). As expected, contraction time is positively correlated with the stimulus inter-spike interval required to reach half the peak tetanic activation (Fig 3E), consistent with a previous experimental observation [70].

Motor units that generate higher motor unit force have faster contraction times (Fig 3D). This association was assumed based on a universal finding that fast-twitch units generate more force than slow-twitch units [147, 148, 159, 170, 171]. We assumed in our default model no correlation between peak tetanic force and contraction time within fiber types (Fig 3D) as a common trophic mechanism does not seem to exist [70].

*Twitch-tetanus ratio*: Twitch-tetanus ratio is computed as the ratio of twitch amplitude to peak tetanic force. This is an important characteristics that influences the activation-frequency relationship of a motor unit and the amplitude of force fluctuations due to unfused tetanic contraction. However, no previous models have explicitly accounted for this and tested its effect on force variability. Here, we assign a twitch-tetanus ratio to each motor unit based on previous experimental observations from cat and rat muscles.

First, the average twitch-tetanus ratio of all motor units of ≈0.23 was determined based on the result from a whole muscle preparation of feline caudofemoralis reported by Brown et al. [139] because the measurement obtained from such preparation is least likely distorted by potential effects of a series-elastic element described above [157]. Then, twitch-tetanus ratios were given to individual motor units such that those values range from 0.07 to 0.53 [104, 155, 162, 164, 166, 168, 172–176] and the ratio within a given fiber-type is positively correlated with contraction time (Fig 3F), as previously reported [166, 167].

### Experimental design and data analysis

#### Simulations of individual motor unit responses

We applied a series of spike trains at various frequencies to isolated, individual motor units without an in-series elastic element to characterize the relationship of discharge rate to activation, force and fusion. The duration of spike trains was set to 3-sec. As in Macefield et al. [151] and McNulty et al. [132], the mean activation (or force) in the last 1-s segment was computed to construct the activation-frequency relationship. To compute the degree of fusion, the peak-to-peak amplitudes, *p*, of the fluctuating activation (or force) signals at the stimulus frequencies were obtained during the last 1-sec segment. The degree of fusion was expressed as a percentage using $(1-\frac{p}{Pt})\cdot 100$, where *Pt* is the twitch amplitude obtained at 1 Hz stimulation [132, 151]. Thus, 0% fusion represents the twitch amplitude and 100% fusion represents no ripples in the activation signals.

#### Simulations of population responses

We ran ten trials at each level of synaptic input (2.5% and 5% and from 10 to 100% with an increment of 10% maximal synaptic input). Each trial consisted of a 1-sec zero input phase, a 2-sec ramp-up and a 13-sec hold at a given level of synaptic input. The last 10-sec of the hold phase was analyzed to quantify mean, standard deviation (SD), and coefficient of variation (CoV) of output force. This segment minimizes potential effects of catch-like properties reported by Leitch and Macefield [177] on the amplitude of force variability. SD and CoV of force were computed using the raw force signals unless noted otherwise. Data are presented as the average of 10 trials in a solid line with the standard deviation indicated with a shaded area.

To characterize the frequency content of force variability, we computed the power spectrum of output force during the last 10-sec of the hold phase using the *pwelch* function in MATLAB, specifying a 0.5 Hz frequency resolution from 0 to 100 Hz without any segmentation or overlap.

We compared the SD-input relationship of motor noise and its amplitude predicted by our new model against those found experimentally by Jones et al. [28] and those assumed in the previous theoretical model by Todorov [12]. The theoretical SD-input relationship (i.e. signal-dependent noise) assumes that standard deviation (SD) of force increases proportionally with mean synaptic input levels (i.e. *SD* ∝ *mean*^*p*^), or

$$\begin{array}{cc} SD=c\cdot mean^{p}, & \end{array}$$ (32)

as described by Jones et al. [28]. The slope, *c*, determines the amplitude of signal-dependent noise and is often fitted to experimental observations to replicate observed kinematic variability [12, 17, 18]. The scaling factor, *p*, equals to 1 for a proportional relationship which has been assumed universally in most models [5–8, 10–14, 16–18].

Jones et al. [28] found the relationship of SD-mean force measured experimentally closely matches the theoretical relationship as in Eq 32. They reported the mean value of *p* across five participants to be 1.05 and SD at 100% MVC to be 2.34%. We derived the value of *c* from Eq 32 with these parameters.

We obtained the value of *c* assumed by Todorov [12] in the following manner. The model by Todorov [12] converts a model input, *u*, into muscle force, *f*, through a second order linear filter using the following equation:

$$\begin{array}{cc} \tau_{1}\cdot \tau_{2}\cdot \ddot{f}\left(t\right)+\left(\tau_{1}+\tau_{2}\right)\cdot \dot{f}\left(t\right)+f\left(t\right)=u\left(t\right). & \end{array}$$ (33)

The time constants, *τ*_1_ and *τ*_2_, are both 40 msec as in Todorov [12]. *u*(*t*) is modeled as *u*_*t*_ ⋅ (1+ *σ*_*c*_ ⋅ *ϵ*_*t*_) where *u*_*t*_ is the input amplitude at time t and *ϵ*_*t*_ ∼ *N*(0, 1). The value of *σ*_*c*_ was set to 0.5 [12]. We varied the value of *u*_*t*_ from 0.1 to 1 with an increment of 0.1 and simulated force output of this system with time step of 10 msec for 15 sec. The standard deviation of force was computed from the last 10-sec segment of the time series. Ten trials were run at each value of *u*_*t*_ and average standard deviation across the ten trials was computed. Finally, we found the slope of the resulting standard deviation-input relationship (i.e. the value of *c*) by fitting a linear equation (*polyfit* function in MATLAB with the degree of 1).

#### Sensitivity analysis

We tested effects of key model parameters on the amplitude of motor noise and its signal-dependence: (1) removing a series-elastic element, (2) increasing the range of peak tetanic force (*PR*), (3) decreasing the range of recruitment thresholds (*U*_*r*_), (4) decreasing the number of motor units (*N*) and (5) changing the recruitment scheme to the conventional onion-skin recruitment scheme and (6) a combination of all. These parameters/features were chosen for the following reasons.

First, as we describe in the Results, addition of a series-elastic element, which has been ignored in previous models (e.g. Moritz et al. [37] and Taylor et al. [39]), can substantially decrease the amplitude of predicted motor noise. The *tibialis anterior* muscle we modeled here has relatively large in-series elasticity as indicated by the tendon+aponeurosis to muscle fiber length ratio [178] of about 4. Small hand muscles commonly used in force variability experiments (e.g. the *first dorsal interosseous* [37, 39] and the *extensor pollicis longus* [28]) have much smaller ratios of around 1-2 [179, 180]. Some larger muscles (e.g. *sartorius* muscle and *deltoid* muscles) have even lower values of about 0.15-0.4 [144, 180–182]. To test the full range of expected in-series elasticity, we chose to remove a series-elastic element from the model (i.e. no in-series elasticity).

Second, Jones et al. [28] noted that the parameters, *PR*, *U*_*r*_ and *N* as key parameters to generate the theoretical SD-force relationship of signal dependent noise. We chose to vary each parameter between the lower and upper range of values reported experimentally. The recruitment threshold of the last recruited unit (i.e. *U*_*r*_) ranges from approximately 0.5 (some intrinsic hand muscles [183]) to 0.8 (TA [46] and biceps brachii [183]). The range of peak tetanic forces is varied from 25-fold (our estimate based on innervation ratio, mean cross-sectional area of muscle fibers, and specific tension) to 100-fold (previously used range based on twitch amplitudes). The lower end of the number of motor units reported experimentally is around 100 from intrinsic hand muscles (e.g. Feinstein et al. [50]).

Finally, our new recruitment scheme was substantially different from those previously used ([30, 37, 131]), potentially biasing our results. Thus, we tested the effect of switching to the conventional onion-skin recruitment scheme. In this recruitment scheme, contraction time of motor units decreases with their size across the entire population. Furthermore, all motor units initiate their discharge at 8 Hz (i.e. *MDR*_*i*_ = 8) and the peak discharge rate (*PDR*_*i*_) of individual motor units progressively decreases from 35 Hz to 25 Hz from the smallest (slowest) motor units to the largest (fastest) motor units. To accommodate these changes in the peak and minimal discharge rates, we modified our recruitment scheme presented above for some motor units whose new *MDR*_*i*_ is higher than *f*_*ti*_ and those whose new *PDR*_*i*_ is lower than 1.1 ⋅ *f*_0.5_. Those units (only one and four units for each case when *N* = 200) were set to increase their discharge rates linearly as in Eq 2. We chose not to use the method originally proposed by Fuglevand et al. [30] where *g*_*e*_ in Eq 3 is constant across all motor units. Based on our preliminary analyses of the model, we found this method can confound our sensitivity analysis because the value of *U*_*r*_ alters the gain of the discharge rate to synaptic input (relative to its maximum) relationship for all motor units, which also influences the expression of motor noise (see more in the Results).

### Statistical analysis

Statistical analysis was performed in the R environment for statistical computing (The R Foundation for Statistical Computing, Vienna, Austria). Details of the statistical analysis performed are given in corresponding results sections.

## Results

The main objective of this study was to re-evaluate the contribution of motor unit properties to force variability (see Introduction, i.e. recruitment and rate coding, the stochastic nature of motoneuron discharge and unfused tetanic contractions).

### New model of a population of motor units improves the simulated behavior of motor units compared to the Fuglevand model

**Fig 4:**
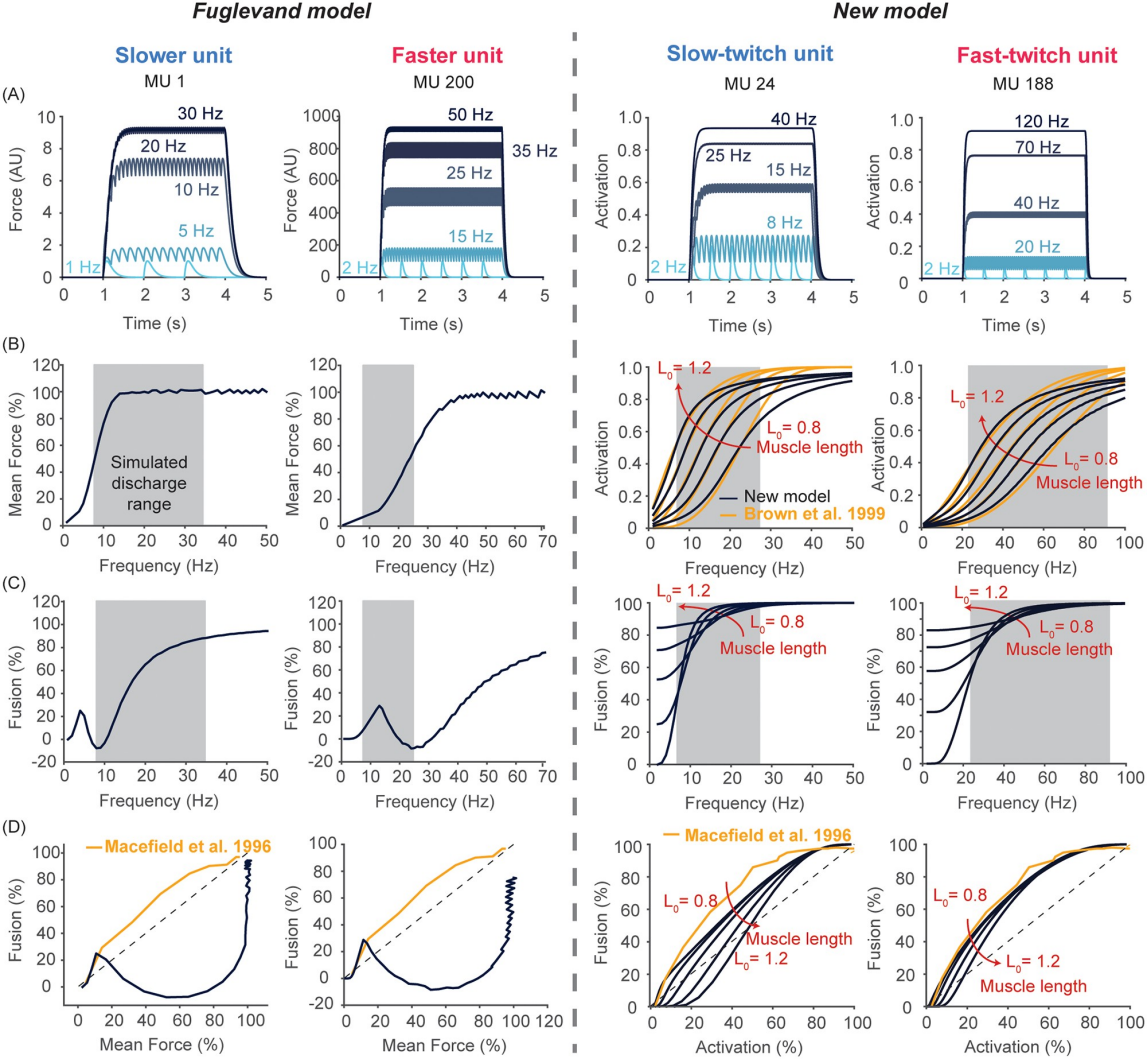
Our new model improves predictions of force production at the individual motor unit level. (Row A) Output of representative motor units, one slower and one faster, from each model to constant synaptic input to their motoneuron at various frequencies. Note the output of the Fuglevand model is force in arbitrary units, whereas that of our model is motor unit activation (0–1) that is then scaled by peak tetanic force to produce force. (Row B) The output-to-frequency relationship of those same motor units. The shaded area represents the range of simulated discharge rates for those motor unit, which for the Fuglevand model does not correspond to the region of the steepest force-frequency relationship. Also, note that our new model includes the length-dependent output-to-frequency relationship described in [66, 138]. Note that the choice of units is not itself related to model validity, but to display the length-dependence of activation in our model, which was described by Brown et al. [138]. (Row C) The degree of fusion as a function of discharge rates. In the Fuglevand model, the increase in fusion is not monotonic and some units do not approach complete fusion. These issues are corrected in our new model. (Row D) The degree of fusion attained as output levels increase. Compared to the experimental observation from [151], the degree of fusion increases too slowly only to rise abruptly at higher outputs in the Fuglevand model, which is corrected in our new model. The dotted identity line is included for reference.

The use of the Fuglevand model often ignores the motoneuron-muscle unit speed match (see the section on *peak and minimal discharge rate* in Methods). We show in the left two columns of Fig 4 characteristics of force outputs from two of the motor units in the original Fuglevand model: one slower unit and one faster unit in terms of their contraction time. Consistent with previous experimental observations [70, 138, 151, 184], the output force of a motor unit follows a sigmodial relationship with increasing discharge rates of the units and faster units require higher discharge rates to achieve their tetanic force (Fig 4B). However, the discharge rates over which the Fuglevand model is used (shaded areas in Fig 4B) are not matched appropriately to their force output. This range is too broad and shifted to the right for slower motor units, and too narrow for faster motor units. The former largely eliminates the effect of rate coding on force output (e.g. MU 1) and the latter truncates it as some units can only reach a fraction of their peak tetanic force (e.g. ca. 60% for MU 200 shown in Fig 4B). This choice of discharge rates has important implications as shown below.

The second limitation of the original Fuglevand model is its inability to exhibit twitch fusion, particularly for faster motor units (Fig 4C and 4D). It is well known that the amplitude of force ripples during unfused tetanic contraction progressively decreases with increasing discharge rates (e.g. Macefield et al. [151] and McNulty et al. [132]). Yet, most motor units in the Fuglevand model do not tend to reach complete fusion (a value close to 100%, see examples in Fig 4C). In some units, the degree of fusion actually *decreases* at intermediate discharge rates—which is not seen experimentally (e.g. [132, 151]). Furthermore, the rate at which the degree of fusion increases with respect to increases in mean force is too slow compared to the known experimental relationship from Macefield et al. [151] (Fig 4D).

In contrast, we assumed in the new model that simulated discharge rates correspond to the steepest region of the output-to-frequency relationship of individual motor units (Fig 4B) based on the previous experimental evidence (e.g. [61, 67, 75]). As expected, motor units with faster contraction times (4^th^ column in Fig 4) require much faster discharge rates to reach their peak motor unit activation and fusion compared to units with slower contraction times (3^rd^ column in Fig 4). These observations are consistent with experimental observations (e.g. [69, 70]). Accordingly, the range of discharge rates changes with the contraction speed of individual motor units. In these ranges, the new model is able to show nonlinear, yet monotonically increasing, fusion (Fig 4C) that closely approximates the fusion vs. mean force behavior described by Macefield et al. [151] (Fig 4D). The discharge rate at which 50% fusion occurs at *L*_0_ in our model corresponds to 0.85 ⋅ *f*_0.5_ (SD = 0.09), which reasonably approximates that reported experimentally in human motor units (e.g. 0.80 by Macefield et al. [151]).

Moreover, the critical addition of a series-elastic element arising from tendon and aponeurosis and the resulting contraction dynamics, which were not included in the Fuglevand model, necessitates modeling the length-dependence of motor unit activation [138]. Thus, we introduced the length-dependence of the activation-frequency relationship of slow and fast-twitch motor units described by Brown et al. [138] and Rack and Westbury [184] (Fig 4B). Due to the length-dependence of motor unit activation, the degree of fusion also demonstrates length dependence (Fig 4C). Such a dependency might be expected from the kinetics of calcium release, diffusion and uptake because the region of thick- and thin-filament overlap becomes longer as the muscle shortens, changing the diffusion distances to regions on the thin-filaments where cross-bridges may form [185]. To best of our knowledge, there exist no experimental studies that have reported length-dependence of fusion and therefore it remains to be validated. However, the effect of length on fusion is relatively small at intermediate discharge rates and does not seem to affect overall findings of our simulation results discussed below.

### Both ‘onion-skin’ and ‘reverse onion-skin’ patterns emerge from our model

**Fig 5:**
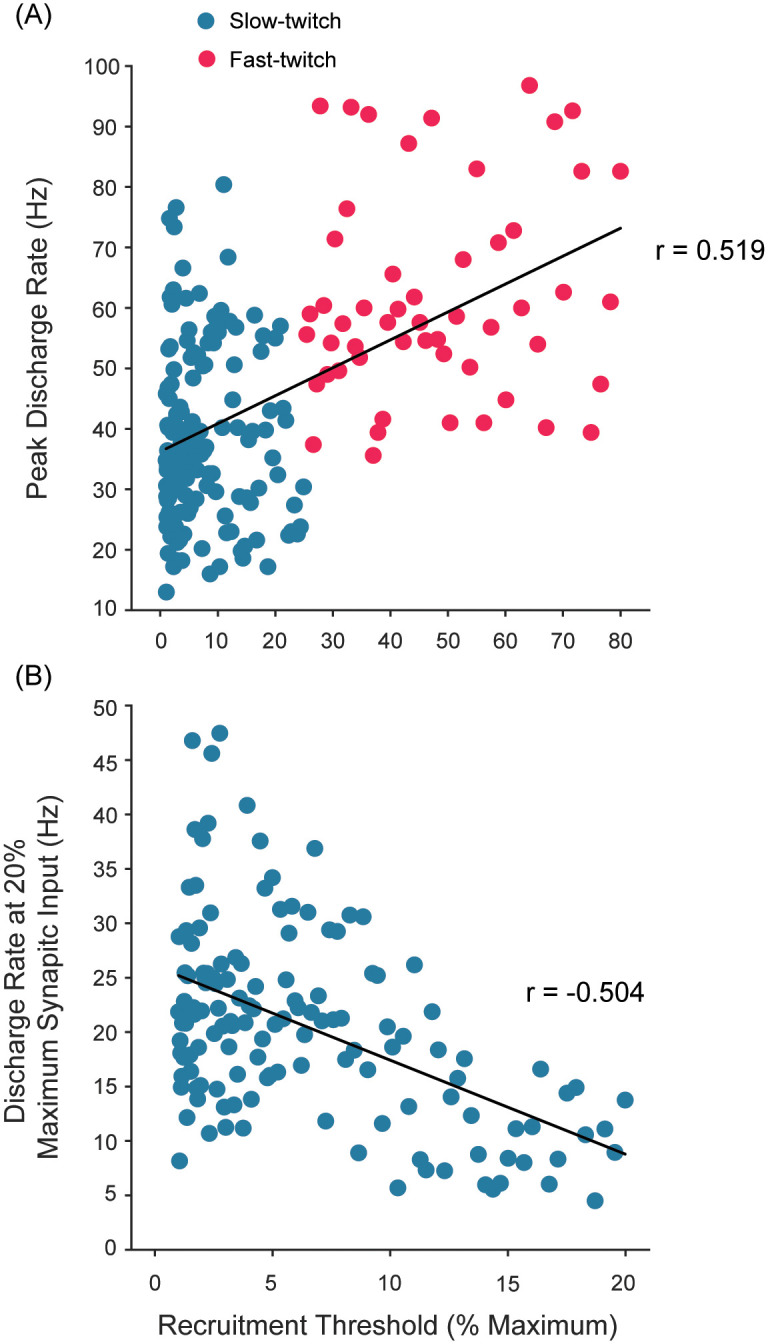
Both ‘onion-skin’ and ‘reverse onion-skin’ patterns emerge from our model. A) The relationship of peak discharge rate vs. recruitment threshold shows a significant positive correlation (r = 0.519, p <0.01). This demonstrates that higher-threshold units tend to show higher peak discharge rates (the reverse-onion scheme feature), without us having explicitly built that in. B) The relationship between the discharge rate at 20% maximum input vs. recruitment threshold shows a significant negative correlation (r = -0.504, p <0.01). In contrast to A), higher-threshold units tend to show slower discharge rates at 20% maximum input, demonstrating the onion-skin pattern as the emergent features of rate coding.

Fig 5A shows the relationship between the peak discharge rate and recruitment threshold of all 200 motor units. We find a significant positive correlation between them (r = 0.519, p <0.01), which is consistent with the previous findings in humans where the recruitment threshold and peak discharge rate were determined during linearly increasing force up to approximately the participant’s maximal voluntary contraction [34, 37, 63, 186]. The same observation applies to the minimal discharge rate in our model (not shown), which is again consistent with previous observations [34, 37, 63, 183, 187]. It is important to note, however, that in our model there exists considerable variability in these relationships because we did not assume a perfect correlation between the size of motor units (and their recruitment thresholds) and the contraction speed within each fiber type (Fig 3D). It is possible to sample pairs of motor units that show opposite relationships (i.e. a higher-threshold unit with lower peak discharge rate)—as is the case experimentally.

Furthermore, we calculated the discharge rate of all active units at intermediate synaptic input levels as done in previous studies [109, 118]. We show a representative result at 20% of synaptic input (*U*_*eff*_ = 0.2) in Fig 5B. When discharge rates of all active motor units are plotted against their recruitment threshold, we can observe a significant negative correlation between the recruitment threshold and discharge rate (*r* = −0.504, p <0.01), which is the characteristic pattern of the onion-skin recruitment scheme ([118, 188–190]).

To further illustrate this point, we randomly sampled 1,000 pairs of motor units from the 19,900 possible pairs in the entire pool (N = 200) and calculated the differences in their recruitment thresholds and in their peak discharge rates. This analysis shows that approximately 65.2% are consistent with the reverse-onion-skin recruitment scheme (i.e. the higher-threshold unit has a higher peak discharge rate) and the balance with the onion-skin recruitment scheme. This was significantly higher than a chance level (p <0.01, binomial test with a probability of 0.5), suggesting that it is more likely to detect the reverse-onion-skin recruitment scheme, but also there is a good chance to find otherwise. Therefore, our model explains why it is not at all surprising that the most experimental studies report conflicting recruitment behaviors [37, 41, 63, 187].

### Unfused tetanic contraction is not the main cause of motor noise

**Fig 6:**
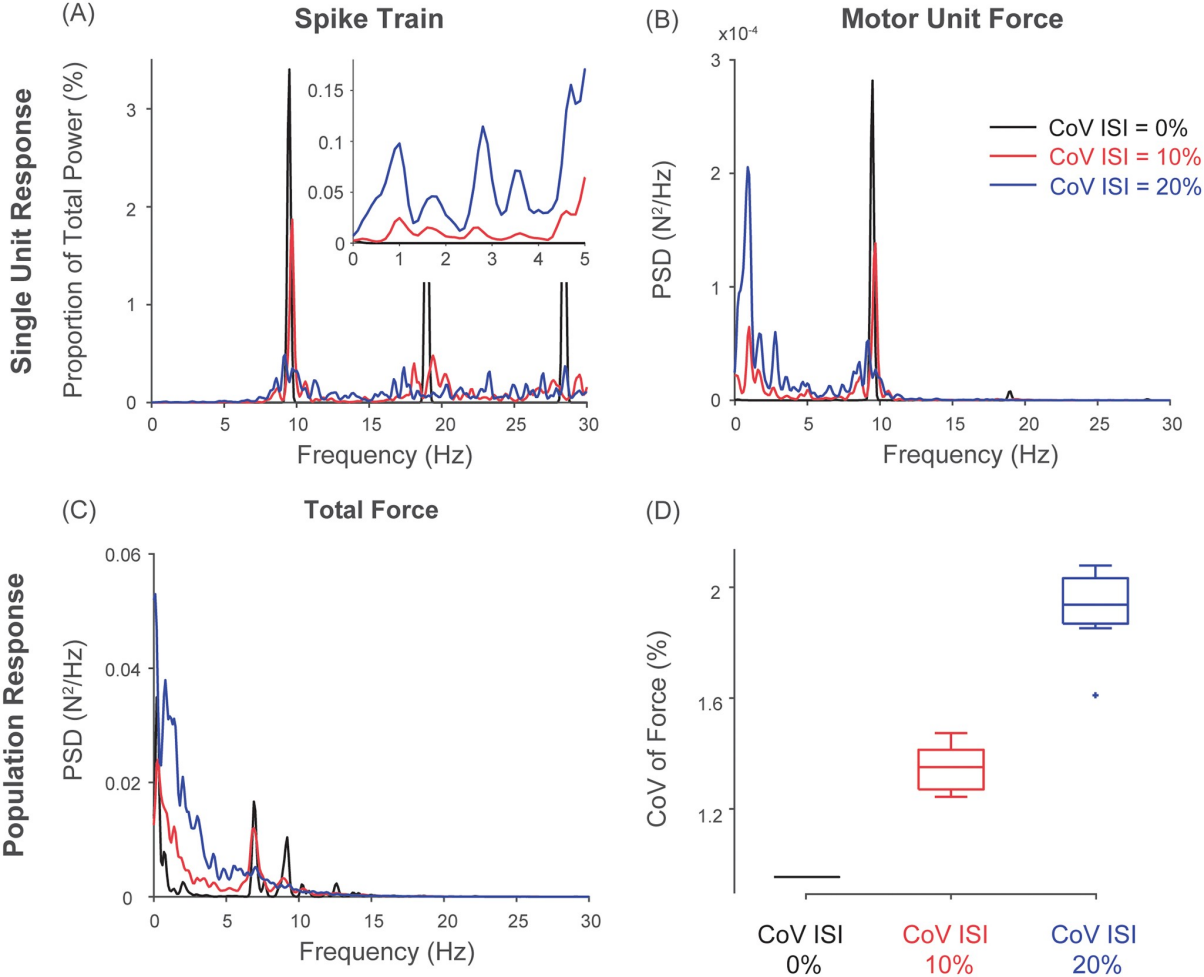
Increased discharge variability causes an increase in force variability through its interaction with the muscle force generating dynamics. A) Power spectral density of a motor unit spike train. Note that increased discharge variability introduces low-frequency (<5 Hz) power. B) Power spectral density of motor unit force. Increased discharge variability increases low-frequency force fluctuations while attenuating those associated with unfused tetanic contraction. C) Power spectral density of tendon force. The spatial filtering of motor unit forces selectively attenuates higher-frequency force fluctuations associated with unfused tetanic contraction. D) CoV of force with varying degrees of discharge variability. Increases in discharge variability result in increases in the overall amplitude of force variability.

At physiological discharge rates, a motor unit produces unfused tetanic contraction [85]. This observation has been used as one of the bases of motor noise [27], yet no previous study has directly quantified its contribution to force variability. Fig 6 shows how the degree of stochasticity, through its interaction with the force generating process of motor units, influence the overall amplitude of force variability. When motor units display no stochasticity (i.e. CoV of ISI = 0%), variability in individual motor unit forces and the total force output of the muscle is solely due to unfused tetanic contractions (Fig 6A, 6B and 6C). However, the contribution of unfused tetanic contractions to the amplitude of motor noise is very limited (Fig 6D). An increase in discharge variability (i.e. an increase in CoV of ISIs) introduces low-frequency power in a spike train of an individual motor unit while attenuating power associated with the discharge rate and its harmonics (Fig 6A). Because the process of converting a spike train into motor unit force acts as a low-pass filter, the low-frequency component (<5 Hz) is accentuated while the higher-frequency counterpart is attenuated (Fig 6B). Furthermore, spatial filtering through summation of motor unit forces across the motor unit population can effectively attenuate the high-frequency force fluctuations (i.e. unfused tetanic contraction), yet its effect is limited for the low-frequency component (Fig 6C). This increase in low-frequency force fluctuations causes the overall amplitude of force variability to increase as the stochasticity in motor unit discharge increases (Fig 6D), which is qualitatively consistent with the experimental data shown by Leitch and Macefield [177] (in their Fig 2). These results demonstrate that unfused tetanic contraction by itself (CoV of ISI = 0% in our simulation) has limited effects on the overall amplitude of force variability. Rather, it is the stochasticity in motor unit discharge that determines the extent to which the activation dynamics due to calcium kinetics and cross-bridge dynamics translates into force variability.

### The Fuglevand model overestimates the contribution of motor unit properties to force variability

**Fig 7:**
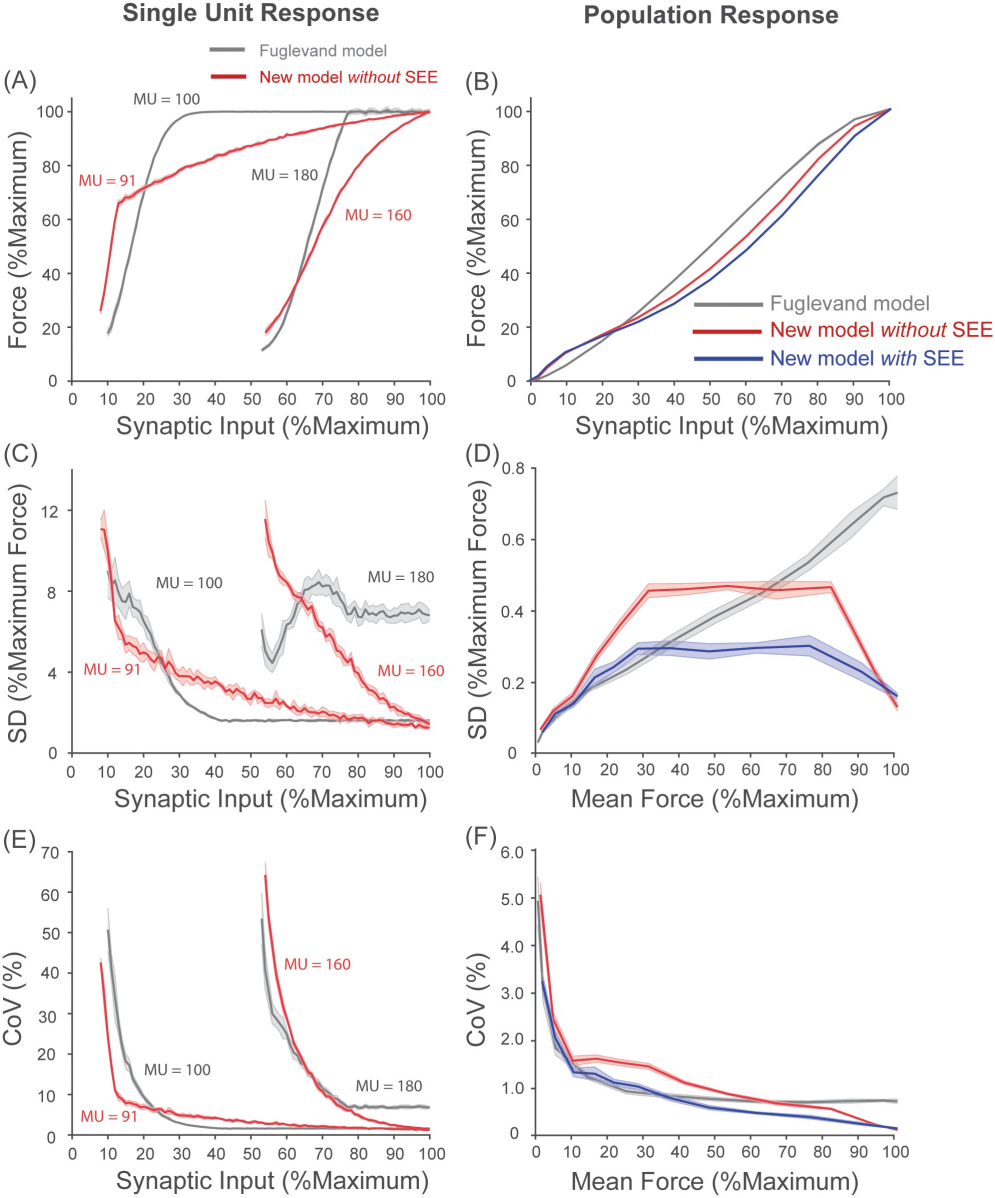
The Fuglevand model can overestimate the contribution of motor noise to force variability. Comparisons between the Fuglevand model and our new model are presented for their single unit responses and population responses. Three simulated conditions are presented as follows: Fuglevand model in gray, the new model *without* a series-elastic element (SEE) in red and the new model *with* SEE in blue. A) Mean force of representative motor units in each model as a function the synaptic input to the entire population. B) Mean force of a motor unit population as a function of the synaptic input. C) SD of force normalized to the maximal force for representative motor units, plotted as a function of the synaptic input to the entire population. D) SD of force normalized to the maximal force for the entire population, plotted as a function of mean force levels. E) CoV of force for representative motor units as a function of the synaptic input to the entire population. F) CoV of force for the entire population as a function of mean force levels. Note that our model no longer displays signal-dependence of SD of force due to progressive decreases in SD and CoV of force in individual motor units. It is important to note that addition of a series-elastic element further reduces the amplitude of motor noise across the entire force levels.

As previously published [28, 35–37, 39–41, 48], the Fuglevand model in our hands also exhibits a monotonic increase in SD of total force throughout the range of force levels (Fig 7D). This signal-dependence of SD on force levels can be attributed to some motor units in the Fuglevand model showing an initial increase in SD of their individual force output and its subsequent plateau (e.g., MU = 180 in Fig 7C). It is important to note that most of the motor units in the Fuglevand model do not show a scaling relationship of *SD* ∝ *mean*^0.47^ shown by Jones et al. [28], suggesting that such a relationship is not a property of motor unit forces but rather an artifact of the model due to its limitations discussed above. More specifically, the initial increase in SD of individual motor unit force output and its subsequent plateau arise from inaccuracy in modeling progressive fusion in the Fuglevand model as our model demonstrates both SD and CoV of individual motor unit forces in all units decrease progressively with the increasing levels of synaptic input (red lines in Fig 7C and 7E). This observation was consistent even when we used a higher value of CoV of ISIs at 20%, which was used by Jones et al. [28]. Accordingly, our model no longer displays the signal-dependence of SD of total force on its mean force levels (Fig 7D). These results suggest that the properties of motor units do not, in and of themselves, suffice to produce signal-dependent noise as was proposed [28] and continues to be accepted [27].

Consistent with our hypotheses, adding physiologically realistic twitch fusion properties greatly reduces SD and CoV of force above 60-70% of the maximal force (red line in Fig 7D and 7F). Addition of a series-elastic element further reduces both SD and CoV of force significantly across the entire range of force levels (Fig 7D and 7F) and amplifies the disparity in the amplitude and signal-dependence of predicted motor noise between the Fuglevand model and our model.

### Musculotendon mechanics are essential for realistic simulation of the spectral characteristics of motor noise

**Fig 8:**
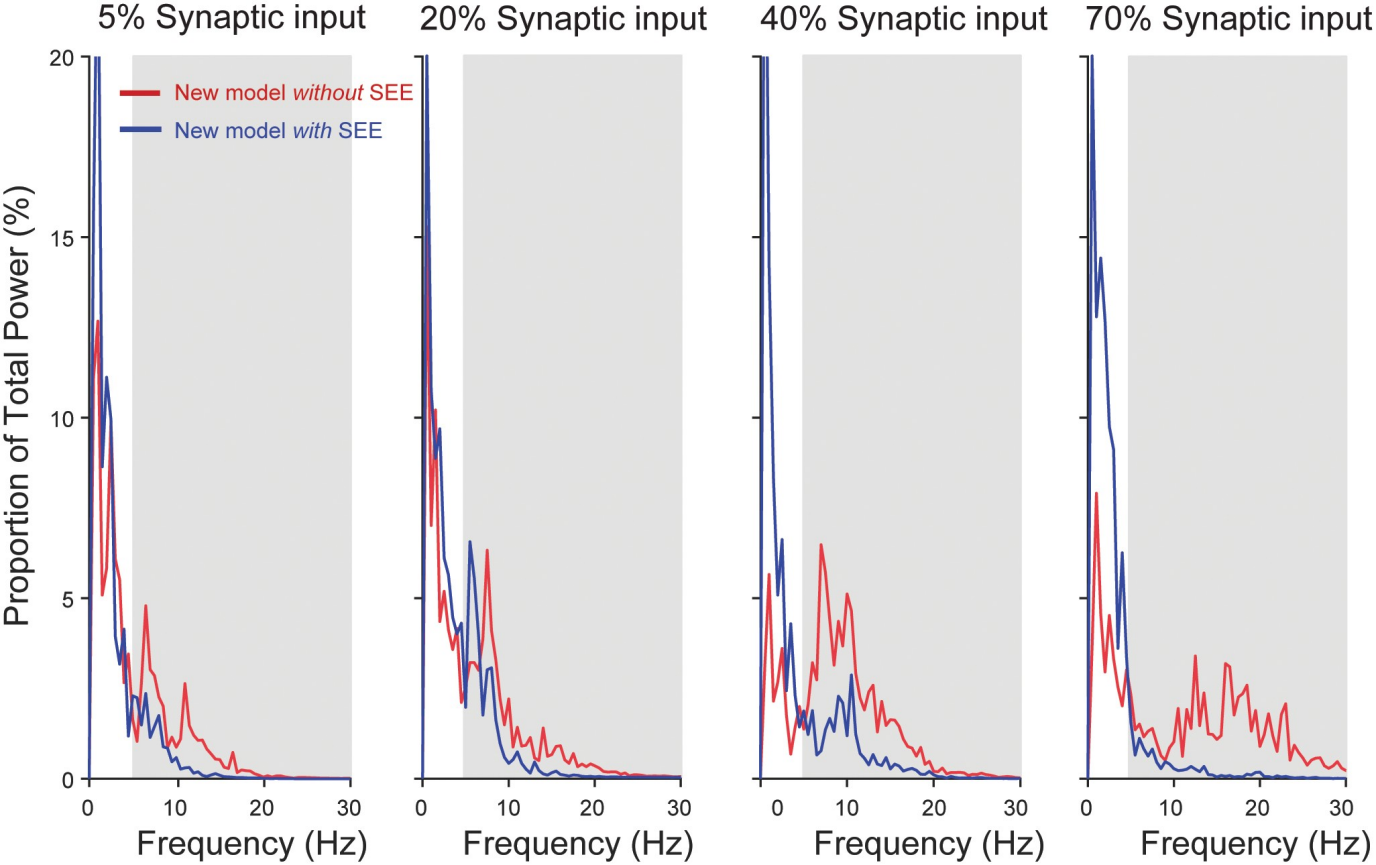
Viscoelastic properties of the contractile element damps high-frequency oscillations associated with discharge rate of motor units. Power spectra of output force at different synaptic input levels (5%, 20%, 40% and 70%) for our new model *without* a series-elastic element (SSE) in red and *with* SEE and blue. Note that addition of SEE substantially reduces power at frequencies >5 Hz (shaded areas).

Addition of the incontrovertible element of a tendon and aponeurosis (i.e. series-elastic element) results in the attenuation of high-frequency (>5 Hz) force fluctuations associated with motor unit discharge (Fig 8). Such attenuation arises from the well-known low-pass filtering effect of the viscoelastic properties of a musculotendon [191–193], yet it has been ignored in, to our knowledge, most previous models of motor units [28, 35–37, 39–41, 48, 194, 195]. This explains smaller CoV for force across the entire force levels shown in Fig 7F.

### Motor noise cannot fully account for the experimentally observed amplitude of force variability or the amplitude of motor noise assumed in previous theoretical models

**Fig 9:**
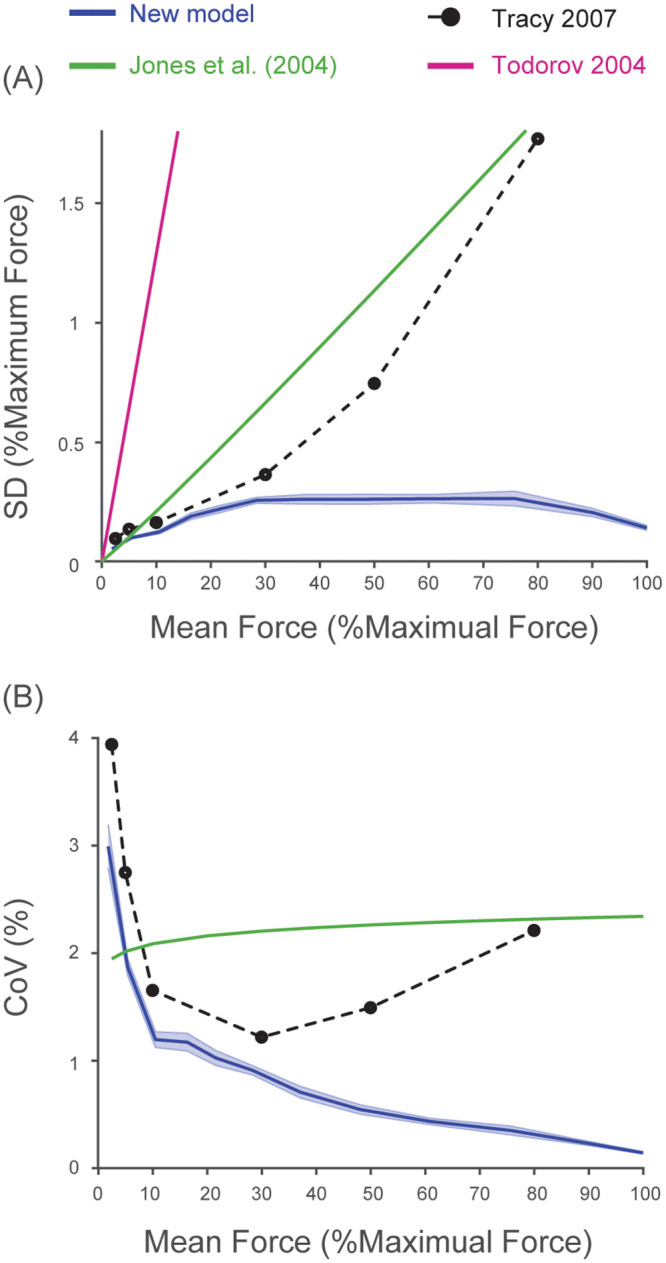
Motor noise cannot fully account for the experimentally observed amplitude of force variability nor for the amplitude of motor noise used in a previous theoretical model. The amplitude of motor noise predicted by our new model is compared to the amplitude of force variability recorded from the *tibialis anterior* muscle in 11 participants reported by Tracy [133]. To allow for a fair comparison between our result and the experimental data by Tracy [133], the 10-sec hold phase of output force was divided into ten 1-sec segments, the duration of data used in Moritz et al. [37] and Tracy [133]. The force signal in each segment was then linearly de-trended using *detrend* function in MATLAB and standard deviation was calculated from the de-trended data. CoV was calculated by dividing the standard deviation by the mean force of the original force signal before de-trending. Our prediction is also compared to the SD-mean force relationship observed experimentally by Jones et al. [28] and that assumed in a previous theoretical model by Todorov [12]. Note that the predicted motor noise is smaller than the experimentally measured force variability (a black dotted line) for the entire possible range of force levels. Our prediction deviates substantially from the theoretical SD-force relationship of motor noise observed experimentally by Jones et al. [28] (a green line) and that implemented in a previous models by Todorov [12] (a magenta line). Experimental data presented adapted from Fig 4A in Tracy [133].

Fig 9A and 9B show that the predicted motor noise by our new model is well below the amplitude of force variability observed experimentally from the *tibialis anterior* muscle by Tracy [133] (the black dotted line) across the entire possible range of force levels. It is worth mentioning that motor noise seems to contribute to higher CoV of force at very low forces demonstrated in the experimental data, which is likely because there are fewer active motor units and lower discharge rates (likely with larger CoV of ISIs as well as we show in S1 Appendix). Note that at these low force levels, the damping effects of the non-linear compliance of tendon and aponeurosis and force-velocity relationship would become much more important, making the actual motor noise much more dependent on muscle architecture (see a large reduction in CoV of force at the lowest force level in Fig 7F).

Furthermore, our prediction highly deviates from the theoretical relationship of signal-dependent motor noise measured experimentally by Jones et al. [28] and that assumed in previous theoretical models: steep, monotonic increases in SD of force (green and magenta lines in Fig 9A). These results altogether suggest that motor noise cannot account for the experimentally observed force variability nor justify the specific implementation of motor noise in many recent theoretical models of motor control.

### Motor unit functional organization is not a primary contributor to signal-dependent noise

**Fig 10:**
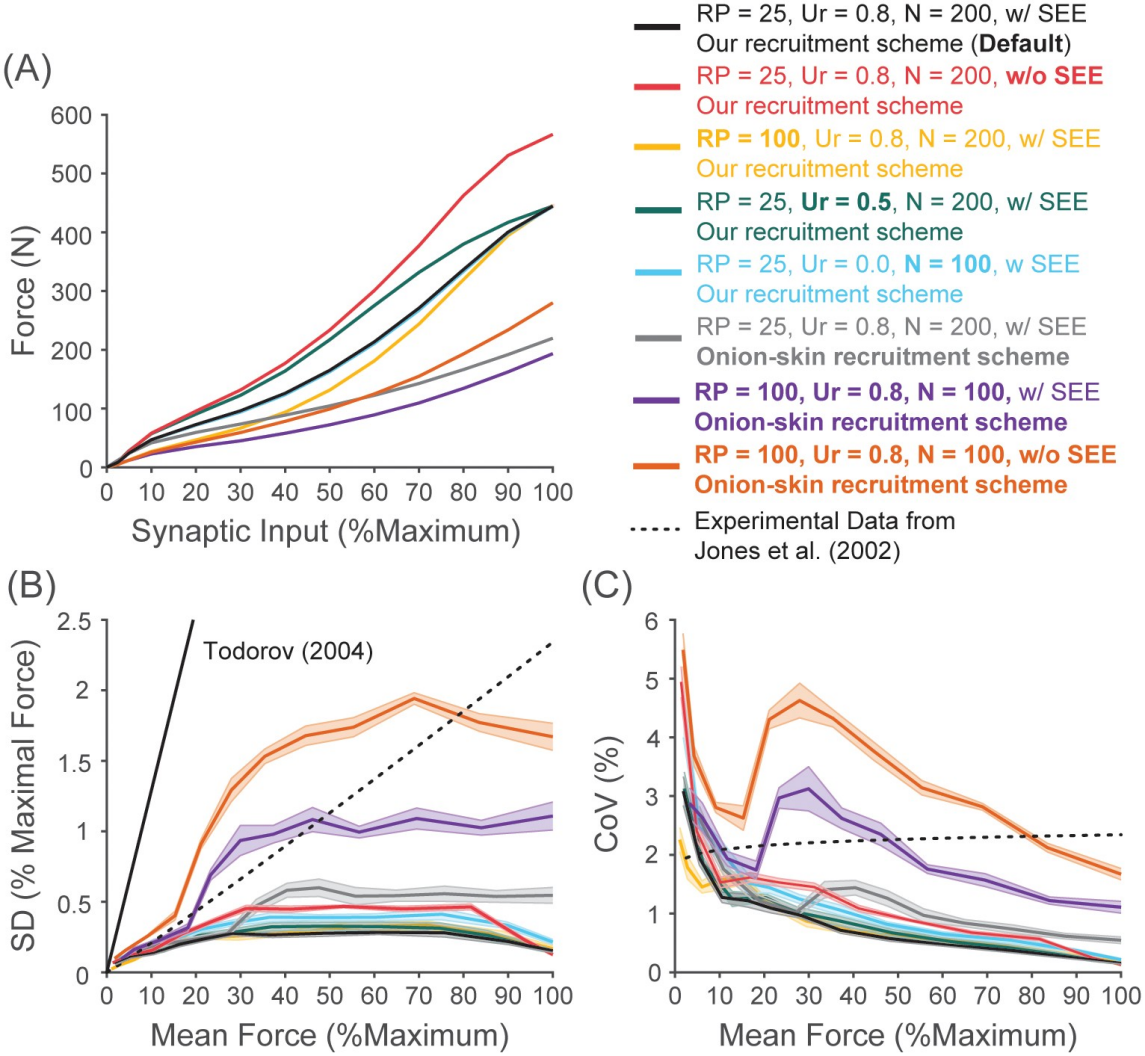
Signal-dependent noise is not the by-product of the motor unit force generation mechanism. We altered several key parameters/features of our model including a series-elastic element (SEE), the range of peak tetanic force (*PR*), the range of recruitment thresholds (*U*_*r*_), the number of motor units in a pool (*N*) and the recruitment scheme (see the figure keys for the detail). To compute SD and CoV for force, we used the last 8-sec of the hold phase. The force signal was divided into two 4-sec segments and each segment was de-trended by a 2^nd^ order polynomial using *detrend* function in MATLAB with an order of 2 as done in Jones et al. [28]. A) Mean force in Newtons as a function of synaptic input levels. B) SD of force (% of maximal force) as a function of mean force levels. A dashed black line represents the SD-mean force relationship observed experimentally by Jones et al. [28]. A solid black line represents the SD-mean force relationship used in the theoretical model by Todorov [12]. C) CoV for force as a function of mean force levels. Despite the large changes in these key parameters/features of our model, predicted motor noise still does not follow the expected SD-mean force relationship and its amplitude is substantially smaller than that used in the previous theoretical model [12].

Fig 10 shows the mean force output of our model as a function of synaptic input levels for various simulated conditions (see the figure keys for detailed descriptions). It is worth noting that an addition of a series-elastic element reduces the maximal force output of the muscle due to shortening of muscle fibers with increasing muscle force applied to the series-elastic element. Also, the conventional onion-skin recruitment scheme (gray, purple and orange lines) substantially reduce the maximal force output because of slower discharge rates (25-30 Hz) of larger and faster motor units, which limit the extent of motor unit activation those motor units can attain (Fig 4).

Most importantly, we demonstrate in Fig 10B that despite the wide range of cases we tested, the relationship of the SD-mean force does not show clear signal dependence reported by Jones et al. [28]. It is worth noting that conditions with the onion-skin recruitment scheme (orange and purple lines) substantially increase the amplitude of predicted motor noise across the entire range of force levels. This is due to the lower discharge rates of larger and faster motor units, causing them to produce unfused tetanic contractions with the very low degree of fusion (Fig 4). These results contrast those reported by De Luca and Contessa [131] demonstrating smaller CoV of force with the onion-skin recruitment scheme compared to the reverse-onion-skin recruitment. It is also important to note that our model is highly *insensitive* to certain parameters such as *PR* and *U*_*r*_ (yellow and green lines) in contrast to the results shown by previous studies using the Fuglevand model [28, 37, 196]. These results suggest that the sensitivity of the Fuglevand model on the parameters, *PR* and *U*_*r*_ in the expression of signal-dependent noise described by Jones et al. [28] is an artifact of its inaccuracy in modeling physiological behavior of motor unit force generation.

The amplitude of motor noise in terms of SD and CoV in all conditions we tested is always substantially lower than that assumed by Todorov [12] in his theoretical model. These results further emphasize our conclusion that the amplitude of motor noise and its signal-dependence assumed in previous theoretical models has no physiological basis in the motor unit force generation mechanisms [5–9, 11–14, 17, 18].

## Discussion

The main objectives of this study were to test the hypotheses that force variability due to motor unit properties (i.e. the stochastic nature of motoneuron discharge and unfused tetanic contraction) is smaller than previously assumed, and is not directly proportional to mean force levels (i.e. signal dependent noise). Our reexamination of the classic Fuglevand model revealed non-physiological properties/assumptions that limit its ability to accurately estimate motor noise. Based on this finding, we have developed a new model of a population of motor units that includes a more complete set of muscle/motor unit properties, namely, 1) calcium kinetics and cross-bridge dynamics that drive fusion of motor unit twitches, 2) coupling between motoneuron discharge rate, cross-bridge dynamics, and muscle mechanics, and 3) a series-elastic element to account for the aponeurosis and tendon. As a result, the updated model disproves common assumptions regarding both the magnitude of force variability accounted for by ‘motor noise’ and its ability to account for the proportional increase in standard deviation (SD) with mean force levels (i.e. *SD* ∝ *mean*^1^) reported in previous experimental studies [28, 31, 37] and implementation of signal-dependent motor noise in previous theoretical models [6, 12, 17, 18].

The key questions using computational models are whether the theoretical structure of a model is founded on the underlying physiological mechanisms and whether such a model can robustly replicate experimental data with realistic ranges of parameters [85, 196–198]. We find that improving the physiological realism of the muscle model, as we report here, explains less of the observed constant variance of force while participants trying to maintain steady force, suggesting that the source of the phenomenon resides in another, as-yet unmodeled part of the system. The mechanisms proposed for motor noise are invariant properties of motoneurons and muscle mechanics, whereas other mechanisms in other parts of the nervous system that appear to be necessary to account for constant variance would likely be subject to many modulatory processes. This begs new models to account for the additional variance. It also provides an opportunity to design experimental paradigms in which variance may not be constant.

### Onion-skin or reverse onion-skin?

The issue of whether the recruitment scheme of a motor unit population follows either the ‘onion-skin’ or ‘reverse onion-skin’ patterns during voluntary contractions in human has been debated over more than 40 years (e.g. [131]). As such, comparisons of model performance between the two recruitment schemes have been standard practice in previous studies (e.g. [30]). Yet, such comparisons do not fundamentally solve the underlying issue because assuming one recruitment scheme over the other leaves some of the experimental data unexplained. There are two main issues in such debate. 1) It has been standard practice to apply an idealized, or simplified, recruitment scheme to a model of a motor unit population that ignores inherent variability in the human motor unit recordings from which the model is constructed. 2) Blindly applying experimental data to a model, even if the model is based on the best available data, lacks an attempt to construct an argument based on the underlying physiological mechanisms/principles. Instead, we demonstrate that our new recruitment scheme built based on a set of fundamental principles of motor unit physiology (see Methods) can display features consistent with both the ‘onion-skin’ and ‘reverse onion-skin’ recruitment schemes (Fig 5). Our results suggest that describing motor unit recruitment schemes as either onion-skin or reverse onion-skin [28, 32, 33, 35–37, 39–41, 48, 189] may be a false dichotomy, and may not be necessary. In fact, picking one scheme a priori can lead to non-physiological motor unit behaviors (Figs 4 and 7). Specifically, onion-skin recruitment can substantially overestimate the significance of motor noise and make the model very sensitive to certain parameters (Fig 10) although it never produced constant CoV in our models.

Furthermore, our approach can not only account for apparently contradicting experimental observations but also generalize to an additional set of experimental observations. We would expect that the correlation between the peak discharge rate and the recruitment threshold would be smaller if the muscle of interest is composed predominantly of one fiber type (Figs 3B and 4D). Consistent with this prediction, Oya et al. [186] showed no correlation between those two variables when they included all motor units recorded from the *soleus* muscle that is composed predominantly of slow-twitch units. Conversely, Moritz et al. [37] found a higher correlation in the *first dorsal interosseous* muscle whose fiber-type composition is approximately 50:50 [199]. Such a difference across muscles cannot be expected from previous recruitment schemes. These observations strengthen the utility of our approach and in turn highlight the issue of previous models resulting from over-simplification/idealization of inherently variable and conflicting experimental data (e.g. [37, 63, 187]).

### Physiological mechanisms of motor noise

The concept of motor noise is based on two experimental observations: unfused tetanic contraction and stochasticity in motoneuron discharges [27]. We demonstrate here that these mechanisms are insufficient to account for even the majority of experimentally observed force variability. First, high-frequency force fluctuations associated with unfused tetanic contraction have limited effects on the overall amplitude of force variability because they are low-pass filtered through 1) the activation dynamics of muscle fibers (i.e. calcium kinetics and cross-bridge dynamics), 2) spatial filtering through summation of motor unit forces and 3) the contraction dynamics of a viscoelastic musculotendon unit (Figs 6, 8 and 10). Furthermore, the activation dynamics of muscle fibers causes progressive fusion of motor unit twitches as the discharge rate increases, resulting in progressive decreases, rather than increases, in the amplitude of motor noise as force levels increase (Fig 7).

All of these results argue against the significance of unfused tetanic contraction in the generation of signal-dependent motor noise when motor units discharge periodically and asynchronously during normal recruitment in most muscles (cf. Fig 3 in [138]). This leaves stochasticity in motoneuron discharges as a potentially more important source of motor noise. Stochasticity in motor unit discharges has been assumed to stem from synaptic noise—random fluctuations in membrane voltage due to stochastic arrivals of action potentials from many excitatory and inhibitory synaptic sources, all superimposed onto a constant synaptic input [200]. Like unfused tetanic contraction, however, stochastic motor unit discharges cannot account for the experimentally observed features of force variability, nor can this mechanism explain the notion of ‘signal-dependent’ noise as a fundamental property of motoneuron activation (S1 Appendix) with which the neural controller needs to contend [6].

Synaptic noise does not follow the relationship with its mean level that has been assumed in prior models (Eq 32). It is the variance of synaptic noise (i.e. square root of standard deviation), not its standard deviation, that increases proportionally to the mean input level (S1(A) Fig and S1 Appendix). Furthermore, the increase in synaptic noise does not necessarily translate into greater discharge variability of motoneurons. In fact, discharge variability due to synaptic noise actually decreases as the mean synaptic input level (and synaptic noise) increases (S1(C) Fig and S1 Appendix). This prediction is consistent with experimental observations in humans [37, 190, 201].

Synaptic noise might cause high discharge variability due to sporadic discharges of near-threshold units (S1(C) Fig and S1 Appendix), but such discharges would be likely only at low to mid levels of synaptic input where recruitment thresholds of motor units are close to each other and there exist many below-threshold motoneurons. Therefore, this type of sporadic firing becomes irrelevant at higher synaptic input levels in our simulations, where most units are already recruited. Even at low- to mid-levels of synaptic input, hysteresis in motor unit recruitment (lower synaptic current required to de-recruit than recruit a motor unit) can prevent sporadic discharge (S1(C) Fig and S1 Appendix) and promote self-sustained discharge [202]. Low threshold units, which are most susceptible to sporadic discharge, are the most strongly affected by hysteresis-generating plateau potentials [128, 129]. In fact, recordings of single motor units in behaving animals and humans (e.g. Fig 1 in Baweja et al. [203] and Figs 1 and 4 in Broman et al. [204]) rarely if ever show sporadic discharges.

Finally, simultaneous changes in the muscle contraction speed would likely compensate for time-dependent changes in motoneuron discharge rates during constant voluntary activation (e.g. firing rate adaptation and fatigue). For example, decreases in the discharge rate of fast-twitch units during a fatiguing contraction are often accompanied by slowing of muscle contraction speed (prolongation of twitch rise- and fall-times), which increases the degree of fusion at a given discharge rate [205]. The combination of these changes would likely minimize changes in the amount of motor noise as slowing of muscle contraction speed would increase the degree of fusion at a given discharge rate (Fig 4).

### Alternative sources of force variability: Fluctuating synaptic input due to feedback-driven control

Experimentally observed fluctuations in force variability are not appropriately characterized as random motor noise, as discussed above. It is critical then to revisit physiological mechanisms through which force output is actually controlled.

Although many studies assume that force variability in the absence of visual feedback is solely due to motor noise, this assumption is likely invalid. Without veridical feedback, motor output (i.e. force or discharge rate of a motor unit) tends to drift (e.g. [206, 207]), suggesting that subjects do not maintain a constant level of synaptic input. The direction of force drift is not random, but rather very stereotypical and becomes larger as the target force level increases [207]. Importantly, Tracy [133] and Vaillancourt and Russell [208] showed the standard deviation of detrended force stays constant despite the significant amount of force drift over time (reductions of up to 20%MVC as in Fig 2B of Vaillancourt and Russell [208]). This observation directly contradicts with the notion of signal-dependent noise as we would expect to see a decrease in the standard deviation of force as the force continues to drift downward. This is likely because another active control mechanism, probably based on memory and somatosensory information [133, 208], is being used in an attempt to maintain a constant force level. The importance of such a control mechanism is highlighted by the much larger extent of force drift in patients with Parkinson’s disease in the absence of visual feedback [209]. These previous observations suggest that there remains an yet unmodeled control mechanism that could explain the difference between our predicted motor noise and experimentally observed force variability in the absence of visual feedback (Fig 9).

As a consequence of the natural tendency of force to drift in the absence of veridical feedback, maintaining output at a constant level requires closed-loop control via sensory feedback (e.g. visual, auditory, etc.). Because of the inherent delay in our sensorimotor systems, closed-loop control introduces low-frequency (≈1-2 Hz) fluctuations in synaptic drive and resulting muscle force [21, 29, 203, 207, 210]. Similarly, proprioceptive feedback modulates synaptic inputs in the 5-12 Hz range due to its interactions with musculotendon mechanics [23]. Furthermore, descending inputs contain oscillations from distinct sources, such as the alpha-band (8-15 Hz) arising from the subcortical structures (e.g. [211–213]) and the beta-band (15-30 Hz) and gamma-bands (35-70 Hz) arising from the cortical structures (e.g. [214, 215]). These oscillations from various sources may also add to or modulate the low-frequency (< 5Hz) component of synaptic drive/force [23, 216]. Failure to consider these structured, dynamic fluctuations in synaptic input leaves discharge variability to be accounted for erroneously as the consequence of synaptic noise [217] and modeled as a constant, random process.

An additional consequence of an inevitably fluctuating level of synaptic input is that these fluctuations are often shared across the pool of motor units and cause synchronization among them [218]. Correlated motor unit discharges are expected to have disproportionately large effects on force variability compared to asynchronous motor unit discharges. The effects of this would be largest below 10 Hz [218–220] due to the low-pass filtering properties of motor units (Fig 7, cf. [221]). Accordingly, studies have found the degree of motor unit synchronization below 5 Hz to be correlated with the amplitude of force variability [222–224]. Increased force variability due to aging [222, 224] and neurological conditions such as stroke [225] and essential tremor [226] have been associated with increases in this low-frequency force variability. Furthermore, a recent study showed that the degree of synchronization below 5 Hz increases with contraction intensity [227], suggesting that increases in force variability at higher forces arise from increased low-frequency fluctuations in the common synaptic inputs (rather than increased motor noise), which are probably due to shifts in high-level control in compensation for drift or fatigue [207]. Finally, it is important to mention that, although synchronized activity above 5 Hz has limited effects on the overall amplitude of force variability in healthy neuromuscular systems, many neurological disorders have been associated with amplified synchronization/force variability above 5 Hz (e.g. [226, 228–230].

Common synaptic inputs can also cause pairs of motor units to discharge nearly simultaneously within approximately 10 ms of each other (short-term synchronization/synchrony, Sears and Stagg [231]). However, experimental data argue that its effect is limited [232], although a simulation study has suggested otherwise [40]. Furthermore, the strength of short-term synchronization can differ quite drastically across muscles and even in the same muscles across tasks [233]. These observations highlight a need for further investigation of the neural mechanisms of short-term synchronization and its consequences on motor control.

### Theoretical and clinical implications

Our findings argue for a reinterpretation of both motor noise and its impact on motor control and learning. We suggest that most muscle force variability and the resulting kinematic variability reflect properties of control strategies embodied through distributed sensorimotor systems. To the best of our knowledge, there is no theoretical model that disambiguates the contribution of control strategies from signal dependent motor noise in observed force/kinematic variability. Rather, many theories based on optimal control (e.g. [6, 12, 17]), as the name suggests, argue that human behavior is an implementation of ‘optimal control’ corrupted by additive noise of arbitrary origin, leaving no room for force variability arising from any other source. Harris and Wolpert [6] proposed a theory that stereotypical kinematic profiles of human reaching movement and saccades as well as the speed-accuracy trade-off (i.e. Fitt’s law [234]) arise from a control strategy attempting to maximize the end point precision assuming the presence of signal-dependent motor noise (the minimum variance theory). However, our results show that their argument is based on a non-physiological assumption, calling into question the validity of their theory.

An important corollary to this revised interpretation for observed variability is that kinematic variability may not necessarily reflect the performance limitation imposed by the motor system as proposed by optimal control theories [6, 12, 17, 18], but rather implies the tendency of the brain to use non-optimal, ‘habitual’ control strategies [235, 236]. This is consistent with experimental observations that various sensorimotor control circuits which influence performance variability may be tuned as needed (as per the precision requirements of a task or different portions of a task) or when useful (e.g. purposeful exploration, persistence of excitation and active sensing) [1, 4]. Rather than lumping such variability into a single coefficient of variation, spectral analyses of variability should help to understand both the underlying mechanisms and their alterations after disease or injury. The experimentally observed tendency of the nervous system to maintain a constant total variance of force is itself in need of explanation. We suggest that force variability is a rich source of information about how the nervous system executes voluntary action; it is mostly not the result of constant, low-level, random noise.

## Supporting information

S1 Fig Our motoneuron model demonstrates that synaptic input noise does not account for signal dependent noise in synaptic input. A) The relationship between mean synaptic current and its standard deviation (SD) generated by asynchronous, random synaptic inputs. Note that this relationship is expressed as *SD* ∝ *mean*^0.5^. B) Motoneuron discharge rates as a function of synaptic currents with (blue line) and without hysteresis (orange line) in motoneuron discharges. As expected, the injection of PIC of 1*nA* (orange line) increases discharge rates. C) Discharge variability of a motoneuron as a function of synaptic currents with (blue line) and without hysteresis (orange line). Note that increasing the amount of synaptic current causes dramatic decreases in CoV of ISIs with increasing levels of synaptic current even in the absence of hysteresis. Note also that hysteresis significantly reduces CoV of ISIs at lower levels of synaptic currentß.
(TIF)

S1 Appendix Analysis of synaptic noise. (PDF)
