# Supplementary figures and images for: Force variability is mostly not motor noise: Theoretical implications for motor control
Source: PLoS Comput Biol. 2021 Mar 8;17(3):e1008707. doi: 10.1371/journal.pcbi.1008707 (PMC7971898; doi:10.1371/journal.pcbi.1008707)

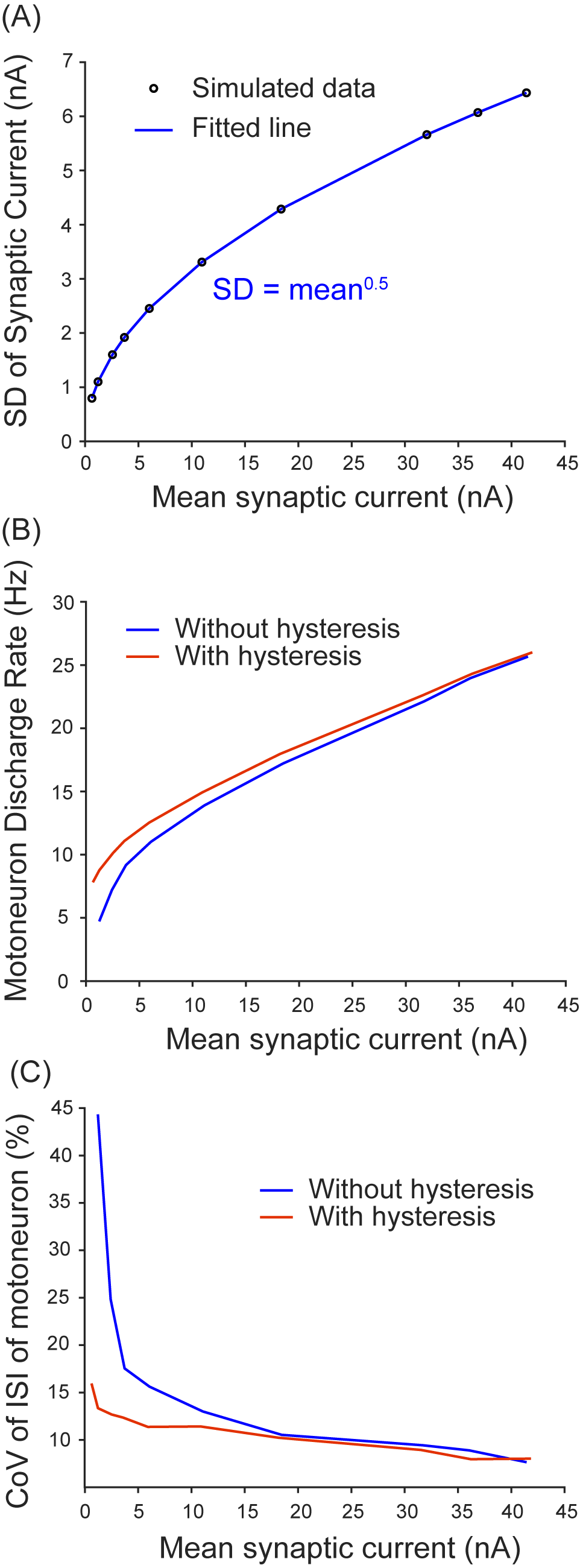

Supplement: S1 Fig — A) The relationship between mean synaptic current and its standard deviation (SD) generated by asynchronous, random synaptic inputs. Note that this relationship is expressed as SD ∝ mean0.5. B) Motoneuron discharge rates as a function of synaptic currents with (blue line) and without hysteresis (orange line) in motoneuron discharges. As expected, the injection of PIC of 1nA (orange line) increases discharge rates. C) Discharge variability of a motoneuron as a function of synaptic currents with (blue line) and without hysteresis (orange line). Note that increasing the amount of synaptic current causes dramatic decreases in CoV of ISIs with increasing levels of synaptic current even in the absence of hysteresis. Note also that hysteresis significantly reduces CoV of ISIs at lower levels of synaptic currentß. (TIF) [file pcbi.1008707.s001.tif]
