# Supplementary material for: Force variability is mostly not motor noise: Theoretical implications for motor control
Source: PLoS Comput Biol. 2021 Mar 8;17(3):e1008707. doi: 10.1371/journal.pcbi.1008707 (PMC7971898; doi:10.1371/journal.pcbi.1008707)
Supplement: S1 Appendix — (PDF) [file pcbi.1008707.s002.pdf]

# Appendix

Table A: **Model parameters for the motoneuron model:**

Our conclusions may raise an alternative, simplistic explanation that signal-dependent variability in force output requires signal-dependent noise in synaptic input to a motor unit population, as previously postulated [1]. In this Appendix, we explore this potential explanation in detail.

The most plausible, if not the only, source of noise in synaptic input is the so called synaptic noise: apparently random membrane voltage fluctuations in motoneurons [2]. The predominant source of synaptic noise is thought to be synaptic bombardment by many asynchronous, stochastic excitatory and inhibitory inputs [2]. However, we demonstrate here that synaptic noise and the resulting motoneuron discharge variability do not display statistical properties required for signal-dependent noise.

## Model of motoneuron membrane dynamics

We developed a motoneuron model using a pulse-based approximation of the Hodgkin-Huxley model of a neuron originally proposed by Destexhe [3] and extended to a two-compartment model of a motoneuron by Cisi & Kohn [4]. The membrane potentials of the dendritic ($V_d$) and somatic ($V_s$) compartments of the model are described by the following system of coupled differential equations using the parameters in Table A:

$$C_d \frac{dV_d(t)}{dt} = I_{eff}(t) - g_{ld}(V_d(t) - E_l) - g_{cd}(V_d(t) - V_s(t)) \quad (1)$$

$$C_s \frac{dV_s(t)}{dt} = -I_{ion}(t) - g_{ls}(V_d(t) - E_l) - g_{cs}(V_s(t) - V_d(t)) \quad (2)$$

$$I_{ion}(t) = \overline{g}_{Na} m(t)^3 h(t)(V_s(t) - E_{Na}) + \overline{g}_{Kf} n(t)^4 (V_s(t) - E_K) + \overline{g}_{Ks} q(t)^2 (V_s(t) - E_K), \quad (3)$$

where

$$C_d = C_m \cdot A_d \tag{4}$$

$$C_s = C_m \cdot A_s \tag{5}$$

$$g_{ld} = A_d/R_d \tag{6}$$

$$g_{ls} = A_s/R_s \tag{7}$$

$$g_c = \frac{2}{\frac{R_i l_d}{\pi r_d^2} + \frac{R_i l_s}{\pi r_s^2}}. \tag{8}$$

We used a pulse approximation to simulate the time course of state variables for each conductance, $m$, $h$, $n$, and $q$ with the pulse duration of 0.6 ms as done previously [3, 4]. Before and after the pulse, the unforced dynamics of the state variables are described by:

$$m(t) = m_0 exp(-\beta_M(t - t_0)) \tag{9}$$

$$h(t) = 1 + (h_0 - 1)exp(-\alpha_H(t - t_0)) \tag{10}$$

$$n(t) = n_0 exp(-\beta_N(t - t_0)) \tag{11}$$

$$q(t) = q_0 exp(-\beta_Q(t - t_0)), \tag{12}$$

where $t_0$ is the time at which the previous pulse ended and $m_0$, $h_0$, $n_0$, and $q_0$ are the values of those state variables at $t_0$. During the pulse, the forced dynamics of the state variables are described by:

$$m(t) = 1 + (m_0 - 1)exp(-\alpha_M(t - t_0)) \tag{13}$$

$$h(t) = h_0 exp(-\beta_H(t - t_0)) \tag{14}$$

$$n(t) = 1 + (n_0 - 1)exp(-\alpha_N(t - t_0)) \tag{15}$$

$$q(t) = 1 + (q_0 - 1)exp(-\alpha_Q(t - t_0)) \tag{16}$$

where $t_0$ is in this case the time at which the pulse started and again $m_0$, $h_0$, $n_0$, and $q_0$ are the values of those state variables at $t_0$.

| | |
|---|---|
| Rheobase current, $I_r$, $(nA)$ | 1.0 |
| Membrane specific capacitance, $C_m$, $(\mu F/cm^2)$ | 1 |
| Dendrite specific resistance, $R_d$, $(k\Omega cm^2)$ | 15 |
| Soma specific resistance, $R_s$, $(k\Omega cm^2)$ | 0.6 |
| Soma surface area, $A_s$, $(\mu m^2)$ | 5943 |
| Dendrite-soma surface area ratio, $p$ | 38 |
| Sodium conductance, $\overline{g}_{Na}$, $(mV)$ | 30 |
| Fast potassium conductance, $\overline{g}_{Kf}$, $(mV)$ | 4 |
| Slow potassium conductance, $\overline{g}_{Ks}$, $(mV)$ | 167.6 |
| Leakage Nernst potential, $K_l$, $(mV)$ | 0 |
| Sodium equilibrium potential, $K_{Na}$, $(mV)$ | 120 |
| Potassium equilibrium potential, $K_K$, $(mV)$ | -10 |
| $\alpha_M$, $\beta_M$ $(1/ms)$ | 22, 13 |
| $\alpha_H$, $\beta_H$ $(1/ms)$ | 0.5, 4 |
| $\alpha_N$, $\beta_N$ $(1/ms)$ | 1.5, 0.1 |
| $\alpha_Q$, $\beta_Q$ $(1/ms)$ | 1.7, 0.024 |

The resulting motoneuron model displays the following passive and active electrical properties. The input resistance and membrane time constant of this motoneuron model are, respectively, 4.4 MΩ and 9.6 ms, which correspond to the upper end of experimentally observed values [5–8] and may be considered a motoneuron innervating slow-twitch muscle fibers. The parameters associated with after-hyperpolarization are as follows: duration of 202.1 ms, magnitude of 9.1 mV, and half-decay time of 28.0 ms, all of which are within the values for motoneurons that innervate slow-twitch units [8].

## Model of synaptic input and the resulting noise

We simulated synaptic noise expected from bombardment of many asynchronous, stochastic excitatory and inhibitory inputs. Here, we assume that 100 excitatory and inhibitory pre-synaptic neurons synapse onto a motoneuron (an extremely conservative model given that motoneurons are estimated to receive synapses from 10-20,000 independent sources [9]). The activity pattern of those pre-synaptic neurons was modeled as a Poisson process with a given mean discharge rate defined in 1-sec time windows. This method has been suggested as an adequate description of the combined synaptic input from many sources insofar as individual synaptic inputs can be described as a renewal process and are independent [10]. The synaptic current was modeled as excitatory and inhibitory conductances for each synaptic contact as follows:

$$I_{syn}(t) = \sum_{i=1}^{n}(g_{syni}(t)(E_{syni} - V_d(t))), \tag{17}$$

where $g_{syni}$ is the synaptic conductance of the $i$-th presynaptic neuron and $E_{syni}$ is the reversal potential of the $i$-th presynaptic neuron (70 mV for excitatory and -16 mV inhibitory synapses) [4]. The time course of $g_{syni}$ was modeled using the following equation [4, 11]:

$$g_{syni}(t) = g_{max}r(t), \tag{18}$$

where $g_{max}$ is the maximum conductance of the synapse (0.1 $nS$) and $r_t$ describes the time course of conductance change [4, 11]. The same parameters as in [11] were used (i.e., $\alpha = 2\ msec^{-1}$, $\beta = 1\ msec^{-1}$ and $T_{max} = 1\ mM$, cf. Eq.4-5 in Destexhe *et al.* [11]).

## Model of hysteresis in motoneuron channels

The activity of voluntary activated motoneurons tends to show hysteresis in their discharge thresholds (e.g. Gorassini *et al.* [12]). This hysteresis is characterized as lower synaptic current required to de-recruit than recruit a motoneuron [13]. Such hysteresis promotes self-sustained discharges [13] and can prevent sporadic discharges. The primary mechanism of this hysteresis is thought to be plateau potentials due to calcium-mediated persistent inward current (PIC) [13, 14].

Here, we modeled the effect of PIC as a step current superimposed on the effective synaptic current as done in a previous simulation study [15]. We opted for this very simplified implementation of PIC over detailed ones (e.g. Powers & Heckman [16, 17]) because its parameters can be more easily manipulated and its results more easily interpreted, and because it has been shown to be able to replicate hysteresis due to PIC [15]. Furthermore, we made the simplifying assumption that PIC is activated immediately upon recruitment of a motoneuron as done previously [15]. The amplitude of PIC tested here was 1 $nA$. This value was at the lowest end of experimentally reported PIC amplitudes in reduced preparations (1-30 $nA$ Hamm *et al.* [18] and Lee & Heckman [19, 20]).

## Emergent statistical properties of synaptic noise and the resulting motoneuron discharge patterns

The model we created allowed us to quantify the standard deviation of synaptic noise that a motoneuron receives, and the resulting motoneuron discharge variability. We increased the amplitude of input synaptic current by increasing the discharge rate of the excitatory and inhibitory presynaptic neurons from 0.5 Hz to 100 Hz.

We applied such synaptic input for 4 sec, for each discharge rate of presynaptic neurons, and analyzed the last 3-sec segment of the data. We ran 10 trials for each discharge rate and the average values of each outcome variable across the 10 trials are presented in S1 Fig. The relationship between the mean and standard deviation ($SD$) of synaptic input was characterized using the following equation:

$$SD = a \cdot mean^b + c, \tag{19}$$

where the coefficients, $a$, $b$ and $c$, were found using curve fitting in MATLAB. The coefficient $b$ describes the proportionality relationship between these SD and mean, which is mainly discussed below.

S1A Fig shows that the standard deviation of synaptic noise increases proportionally to the square root of the mean synaptic current (i.e. $SD \propto mean^{0.5}$), which is a well known property of stochastic signals [21]. This relationship indicates that an increase in the level of 'noise' with increasing synaptic input is much smaller than the previously assumed relationship (i.e. $SD \propto mean^{1}$) [1, 22, 23]. Furthermore, the stochasticity in motor unit discharges (i.e. CoV of ISIs) in response to synaptic noise exponentially *decreases* with increasing levels of synaptic current despite the increased level of 'noise' the motor unit received (S1C Fig). Such a pattern emerges from a transition from unstable to stable recruitment of a motor unit. At lower synaptic currents, the mean level of the synaptic current is below or around the recruitment threshold. Thus, random fluctuations due to synaptic noise can cause irregular crossings of the threshold and sporadic discharges, which increases discharge variability. Once the mean current level is well above the threshold, synaptic noise can no longer cause such irregular recruitment and therefore discharge timings become relatively stable. Moreover, the greater stochasticity observed at lower synaptic currents can be reduced dramatically by adding hysteresis in motor unit discharges due to PIC (S1C Fig). It is important to note that only a small amount of PIC (i.e. $1nA$) is required to see such a large effect on discharge variability compared to PIC amplitudes observed in reduced preparations [18–20]. This mechanism might explain the sparsity of sporadic discharges in recordings of single motor units in behaving animals and humans (e.g. Fig 1 in [24] and Figs 1 and 4 in [25]). Furthermore, it is interesting to note that the pattern we observed for CoV of ISIs as a function of synaptic current with addition of hysteresis matches more closely the experimentally observed patterns recorded during voluntary contraction in humans (soleus in Fig 12 of [26]).

# References

1. Jones, K. E., Hamilton, A. F. d. C. & Wolpert, D. M. Sources of signal-dependent noise during isometric force production. *Journal of neurophysiology* **88,** 1533–1544 (2002).
2. Calvin, W. H. & Stevens, C. F. Synaptic noise and other sources of randomness in motoneuron interspike intervals. *Journal of Neurophysiolpgy* **31,** 574–588 (1968).
3. Destexhe, A. Conductance-based integrate-and-fire models. *Neural Computation* **9,** 503–514 (1997).
4. Cisi, R. R. & Kohn, A. F. Simulation system of spinal cord motor nuclei and associated nerves and muscles, in a Web-based architecture. *Journal of computational neuroscience* **25,** 520–542 (2008).
5. Fleshman, J. W., Segev, I. & Burke, R. Electrotonic architecture of type-identified alpha-motoneurons in the cat spinal cord. *Journal of Neurophysiology* **60,** 60–85 (1988).
6. Powers, R. K. & Binder, M. D. Distribution of oligosynaptic group I input to the cat medial gastrocnemius motoneuron pool. *Journal of neurophysiology* **53,** 497–517 (1985).
7. Rose, P. & Vanner, S. Differences in somatic and dendritic specific membrane resistivity of spinal motoneurons: an electrophysiological study of neck and shoulder motoneurons in the cat. *Journal of neurophysiology* **60,** 149–166 (1988).
8. Zengel, J. E., Reid, S. A., Sypert, G. W. & Munson, J. B. Membrane electrical properties and prediction of motor-unit type of medial gastrocnemius motoneurons in the cat. *Journal of neurophysiology* **53,** 1323–1344 (1985).
9. Hultborn, H. & Fedirchuk, B. in *Encyclopedia of Neuroscience* 309–319 (Elsevier, 2009).
10. Burkitt, A. N. A review of the integrate-and-fire neuron model: I. Homogeneous synaptic input. *Biological cybernetics* **95,** 1–19 (2006).
11. Destexhe, A., Mainen, Z. F. & Sejnowski, T. J. An efficient method for computing synaptic conductances based on a kinetic model of receptor binding. *Neural computation* **6,** 14–18 (1994).
12. Gorassini, M., Yang, J. F., Siu, M. & Bennett, D. J. Intrinsic activation of human motoneurons: possible contribution to motor unit excitation. *Journal of neurophysiology* **87,** 1850–1858 (2002).

13. Binder, M. D., Powers, R. K. & Heckman, C. Nonlinear input-output functions of motoneurons. *Physiology* **35,** 31–39 (2020).

14. Johnson, M. D., Thompson, C. K., Tysseling, V. M., Powers, R. K. & Heckman, C. J. The potential for understanding the synaptic organization of human motor commands via the firing patterns of motoneurons. *Journal of neurophysiology* **118,** 520–531 (2017).

15. Revill, A. L. & Fuglevand, A. J. Effects of persistent inward currents, accommodation, and adaptation on motor unit behavior: a simulation study. *Journal of neurophysiology* **106,** 1467–1479 (2011).

16. Powers, R. K. & Heckman, C. J. Contribution of intrinsic motoneuron properties to discharge hysteresis and its estimation based on paired motor unit recordings: a simulation study. *Journal of neurophysiology* **114,** 184–198 (2015).

17. Powers, R. K. & Heckman, C. J. Synaptic control of the shape of the motoneuron pool input-output function. *Journal of neurophysiology* **117,** 1171–1184 (2017).

18. Hamm, T. M., Turkin, V. V., Bandekar, N. K., O'Neill, D. & Jung, R. Persistent currents and discharge patterns in rat hindlimb motoneurons. *Journal of neurophysiology* **104,** 1566–1577 (2010).

19. Lee, R. & Heckman, C. Paradoxical effect of QX-314 on persistent inward currents and bistable behavior in spinal motoneurons in vivo. *Journal of Neurophysiology* **82,** 2518–2527 (1999).

20. Lee, R. & Heckman, C. Bistability in spinal motoneurons in vivo: systematic variations in persistent inward currents. *Journal of neurophysiology* **80,** 583–593 (1998).

21. Tkachenko, N. V. *Optical spectroscopy: methods and instrumentations* (Elsevier, 2006).

22. Harris, C. M. & Wolpert, D. M. Signal-dependent noise determines motor planning. *Nature* **394,** 780 (1998).

23. Todorov, E. & Jordan, M. I. Optimal feedback control as a theory of motor coordination. *Nature neuroscience* **5,** 1226 (2002).

24. Baweja, H. S., Patel, B. K., Martinkewiz, J. D., Vu, J. & Christou, E. A. Removal of visual feedback alters muscle activity and reduces force variability during constant isometric contractions. *Experimental brain research* **197,** 35–47 (2009).

25. Broman, H., De Luca, C. J. & Mambrito, B. Motor unit recruitment and firing rates interaction in the control of human muscles. *Brain research* **337,** 311–319 (1985).

26. Matthews, P. Relationship of firing intervals of human motor units to the trajectory of post-spike after-hyperpolarization and synaptic noise. *The Journal of physiology* **492,** 597–628 (1996).
